# Response of gonadotropin‐releasing hormone neurons from female mice to dynamic‐clamp‐simulated GABAergic conductances across development and after prenatal androgenization

**DOI:** 10.1111/jne.70144

**Published:** 2026-02-06

**Authors:** Jennifer Jaime, R. Anthony DeFazio, Suzanne M. Moenter

**Affiliations:** ^1^ Department of Molecular and Integrative Physiology University of Michigan Ann Arbor Michigan USA; ^2^ Department of Internal Medicine University of Michigan Ann Arbor Michigan USA; ^3^ Department of Obstetrics & Gynecology University of Michigan Ann Arbor Michigan USA; ^4^ Department of the Reproductive Sciences Program University of Michigan Ann Arbor Michigan USA; ^5^ Present address: Department of Pediatrics University of Colorado, Anschutz Medical Campus Aurora Colorado USA

**Keywords:** dynamic clamp, GnRH, PCOS, GABA

## Abstract

Disrupted gonadotropin‐releasing hormone (GnRH) secretion patterns can impair fertility as in polycystic ovary syndrome (PCOS).We used prenatally androgenized (PNA) female mice, which recapitulate neuroendocrine abnormalities observed in PCOS patients, to study how changes in GnRH neuron intrinsic properties during development (prepubertal 3‐week‐old versus adult females) and with PNA treatment shape their postsynaptic response to GABAergic input. The properties of isolated GABAergic postsynaptic currents in GnRH neurons were used to generate representative model conductances of 1, 2, 5, and 10 nS, with decay time constants representing prepubertal and adult mice (7 vs. 10 ms). These conductances were applied to GnRH neurons from each experimental group using dynamic clamp, and response was measured. Neither development nor PNA altered the response of GnRH neurons to small conductances (1 or 2 nS), and these conductances did not initiate action potentials. In response to the 5 nS conductance, dynamic‐clamp‐induced postsynaptic potentials were larger in 3‐week‐old controls versus 3‐week‐old PNA mice at the 7 ms decay time constant and larger than vehicle‐treated (VEH) adults at the 10 ms decay time constant. In response to larger conductances, only seven of 78 GnRH neurons from adults generated action potentials, whereas 14 of 73 GnRH neurons from 3‐week‐old females did. Interestingly, an altered action potential waveform was observed only in 3‐week‐old PNA females. The changes in GnRH neuron intrinsic properties occurring with development and PNA treatment result in differential responses to the same physiologic GABA input and may contribute to the action potential firing changes previously reported in this model.

## 1 INTRODUCTION

Distinct patterns of gonadotropin‐releasing hormone (GnRH) secretion from neurons during the female reproductive cycle are critical for fertility.^1^ During the cycle, shifts from low‐ to high‐frequency GnRH pulses help drive the differential release of follicle‐stimulating hormone (FSH) and luteinizing hormone (LH) from the anterior pituitary.^2^, ^3^ FSH and LH regulate ovarian follicle maturation and steroidogenesis, and steroid feedback regulates GnRH and pituitary hormones. Disruptions in these hormone patterns can impair fertility, as in polycystic ovary syndrome (PCOS).^4^, ^5^, ^6^, ^7^, ^8^, ^9^, ^10^ Hyperandrogenemia PCOS patients (8%–10% of women) have persistent high‐frequency LH, and presumably GnRH, pulses.^11^ The etiology of PCOS is unknown but PCOS‐like symptoms, such as hyperandrogenemia and menstrual irregularities, have been reported in peripubertal girls,^12^, ^13^ suggesting that disruptions to the reproductive neuroendocrine system can manifest before reproductive maturation is complete.

Prenatally androgenized (PNA) animal models recapitulate many neuroendocrine aspects of PCOS and have been used to understand central changes.^14^, ^15^, ^16^, ^17^, ^18^ PNA mice exhibit altered reproductive cyclicity, high‐frequency LH pulses, and elevated serum testosterone concentrations.^18^, ^19^ PNA treatment alters GnRH neuron physiology. For example, in VEH females, the spontaneous firing rate of GnRH neurons peaks at 3 weeks of age before falling to lower levels in adulthood.^20^ In contrast, the spontaneous firing rate of GnRH neurons in PNA females does not change throughout development; the firing rate of GnRH neurons from PNA females is thus lower than controls at 3 weeks of age, but higher in adulthood.^17^, ^20^ PNA treatment also increases the frequency of GABAergic neurotransmission to GnRH neurons at all ages studied.^18^, ^21^ Adult GnRH neurons maintain high intracellular chloride concentration and activation of GABA_A_‐receptors depolarizes these cells and can result in action potential firing.^22^, ^23^ The low rate of spontaneous GnRH neuron firing in 3‐week‐old PNA compared to 3‐week‐old VEH females^20^ was initially surprising given that PNA females receive higher frequency GABAergic neurotransmission, but the firing response to locally applied GABA is blunted in 3‐week‐old PNA females despite no difference in baseline potential or chloride reversal potential.^21^ This suggests changes in other GnRH neuron intrinsic properties, such as voltage‐gated channels, input resistance and capacitance contribute to the blunted response of PNA females to GABA at this age. GnRH neurons from adult females are more excitable than those from 3‐week‐old females and the maximum amplitude of the after‐hyperpolarization potential (AHP) is larger and delayed. PNA had no effect on these parameters.^24^ The properties of both a transient and a residual potassium current are altered with age and/or PNA treatment.^25^ A preliminary report suggests calcium current density is increased by PNA treatment at both ages.^26^

How intrinsic properties of GnRH neurons alter the response of GnRH neurons to physiologic synaptic inputs is not possible to study with traditional patch‐clamp approaches. This is because conditions used to optimize study of a particular ionic conductance often preclude examining other conductances or action potentials. Here, we use dynamic clamp, a hybrid computational/electrophysiological technique, to test if GnRH neuron intrinsic properties from 3‐week‐old and adult VEH and PNA female mice alter the response of GnRH neurons to simulated GABAergic conductances. This two‐by‐two design allows us to interrogate the mechanisms underlying age‐ and treatment‐related changes in GnRH neuron action potential output.^20^

## 2 MATERIALS AND METHODS

All chemicals were acquired from Sigma‐Aldrich (St. Louis, MO, USA) unless otherwise noted.

### 2.1 Animals

GnRH‐GFP (Tg(Gnrh1‐EGFP)51 Sumo MGI:6158457) mice were bred in our colony.^27^ All mice were provided with water and chow ad libitum; non‐breeders were fed Envigo 2916, breeders Envigo 2919 (Envigo Bioproducts Inc., Madison, WI, USA). Mice were held on a 14:10 light/dark cycle with lights on at 3 AM Eastern Standard Time. To generate PNA mice, female GnRH‐GFP transgenic mice on a C57Bl/6J background and a CD1 female were housed with a C57Bl/6J male. The CD1 dam assists in providing maternal care and nutrition. Females were monitored daily for a copulatory plug (day 1 of pregnancy). On days 16–18 of pregnancy, GnRH‐GFP dams were injected subcutaneously with 225 μg/day of dihydrotestosterone (DHT) in 50 μL sesame oil for PNA treatment or sesame oil for VEH‐treated controls. Litter sizes were adjusted 1 week after birth to <15 pups by culling the CD1 pups (evident from size and coat color) to standardize nutrition. All procedures were approved by the Institutional Animal Care and Use Committee of the University of Michigan.

### 2.2 Verification of PNA phenotype

Electrophysiological studies were done using brain slices from 3‐week‐old (18–21 days) prepubertal or adult (96–169 days) diestrous females with cycle stage determined by vaginal cytology and confirmed with uterine mass. PNA phenotype was examined directly in animals studied as adults; PNA phenotype was examined for prepubertal mice in littermates raised to adulthood as it is consistent among littermates. PNA effects were measured by monitoring age of vaginal opening (VO), estrous cyclicity via vaginal lavage (12 μL saline solution containing unstained cells was examined and photographed immediately after collection) for 14 consecutive days in adulthood and measuring anogenital distance (AGD) at 70–72 days of age (average of three successive daily measures).

### 2.3 Brain slice preparation

All solutions were bubbled with 95% O_2_/5% CO_2_ for at least 15 min before use. Brain slices were prepared 3.5–6.5 h after lights on. Mice were decapitated, and brains removed and placed in ice‐cold sucrose saline containing the following (in mM): 250 sucrose, 3.5 KCl, 26 NaHCO_3_, 10 D‐glucose, 1.25 Na_2_HPO_4_, 1.2 MgSO_4_, and 2.5 MgCl_2_ (350 mOsm). Coronal slices (300 μm) through the hypothalamic region were cut with a Leica VT1200S Microtome (Leica Biosystems, Buffalo Grove, IL, USA). Slices were incubated for 30 min at room temperature (~21°C–23°C) in 50% sucrose saline/50% artificial cerebrospinal fluid (ACSF, containing [in mM]: 135 NaCl, 3.5 KCl, 26 NaHCO_3_, 10 D‐glucose, 1.25 Na_2_HPO_4_, 1.2 MgSO_4_, 2.5 CaCl_2_, 315 mOsm, pH 7.4). Slices were then transferred to 100% ACSF at room temperature for at least 30 min prior to being used for recordings. All recordings were conducted within 1‐6 h of brain slice preparation; no differences among recordings were attributable to time after brain slice preparation. A minimum of five mice from at least five litters were studied per group; up to three recordings were used per mouse.

### 2.4 Recording solutions and data acquisition

Whole‐cell voltage‐clamp recordings were used to measure GABAergic postsynaptic currents (PSCs) in GnRH neurons; the ACSF contained 20 μm D‐APV (Tocris) and 10 μm CNQX (Tocris) to block ionotropic NMDA and AMPA receptors, respectively. Whole‐cell current‐clamp recordings were used to measure the response of GnRH neurons to simulated conductances; the ACSF contained 20 μM D‐APV, 10 μM CNQX, and 100 μM picrotoxin to block ionotropic glutamate and GABAergic receptors. Recording electrodes (2–4 MΩ) were made from borosilicate glass using a Sutter P97 puller (Sutter Instruments) and filled with a solution containing (in mM): 120 K gluconate, 20 KCl, 10 HEPES, 5 EGTA, 0.1 CaCl_2_, 4 MgATP, and 0.4 NaGTP at 305 mOsm, pH 7.2 with NaOH. This solution was based on the native intracellular chloride concentrations in GnRH neurons determined using gramicidin‐perforated‐patch recordings.^21^, ^22^ A 14.5 mV liquid junction potential was negated online before each recording.^28^ During all recordings, brain slices were continuously perfused with bubbled ACSF at 3 mL/min and maintained at 30°C–31°C with an inline‐heating unit (Warner Instruments Model SH‐27B). GFP‐identified GnRH neurons were visualized with infrared differential interference contrast and fluorescence microscopy on an Olympus BX50WI or BX51WI microscope. Recordings were made using an EPC‐10 patch clamp amplifier and a computer running PatchMaster software (HEKA Elektronik). Recordings were acquired at 20 kHz and filtered at 10 kHz. Recording quality and passive properties were monitored in voltage‐clamp mode throughout experiments from the averaged membrane current response to 16 hyperpolarizing voltage steps from −70 mV for GABA PSC recordings and −65 mV for dynamic clamp recordings (5 mV, 20 ms). Bridge balance (90%) was used for all current‐clamp recordings. Data were analyzed using WaveMetrics IgorPro (Sutter Instruments). Only recordings with an input resistance between 410 and 1500 MΩ, stable compensated series resistance of 7.9–30 MΩ, and a stable capacitance (5.7pF–30 pF) were used for analysis.

### 2.5 Properties of isolated GABAergic PSCs in GnRH neurons

The decay time constant of GABAergic PSCs is strongly dependent on the concentration of chloride in the recording pipette.^29^ We thus measured the properties of GABA PSCs onto GnRH neurons using a physiologic chloride concentration (K gluconate solution above) in cells held at −70 mV. Isolated GABAergic PSCs, defined as events with at least a 50 ms between the peaks of adjacent events, were averaged for each cell recorded (5 to 40 isolated events per cell). The amplitude and decay time constant of the averaged PSC were measured. The decay time constant (tau) was estimated using a monoexponential fit (Equation 1) from 80% to 20% of the peak of the normalized, averaged PSC for each cell, using the following equation, where only tau was allowed to vary.

(1) $$I_{\mathrm{PSC}}=-1e^{\left(-\frac{\text{time}}{\mathrm{tau}}\right)}$$

### 2.6 Modelling of the GABAergic postsynaptic conductances

No difference in the amplitude of averaged GABA PSCs was detected among groups recorded with physiologic Cl^−^ concentrations. Thus, a range of conductances within and moderately above the physiological range of GABA PSCs was used (1, 2, 5, 10 nS). In contrast, an age‐dependent increase in decay time constant from 7.4 ± 0.1 ms to 9.9 ± 0.2 ms was detected (two‐way ANOVA, *F*(1,19) = 7.76, *p* = 0.011). Each conductance was thus modeled twice: using a monoexponential decay time constant of 7 ms, representative of females at 3 weeks of age, and of 10 ms, representative of adult females.

### 2.7 Dynamic clamp recordings

Dynamic‐clamp recordings were performed using a Cybercyte V10 (CytoCybernetics, Inc.). The input to the dynamic clamp system is the membrane potential of the cell ($V_{m}$) read from the EPC10 patch‐clamp amplifier in current‐clamp mode; the output from the dynamic clamp system is the computed command current $\left(I_{\mathrm{dc}}\right)$, which drives the current command input of the EPC10 amplifier. The average loop time measured for the system was 21.29 ± 0.03 μs (mean ± SEM). To implement the synaptic conductance model in the dynamic clamp mode, $I_{\mathrm{dc}}$ was calculated from the postsynaptic conductance ($g_{\mathrm{syn}}$) as a function of the time after a trigger and the linear driving force (Equation 2). In the equation below, $E_{\mathrm{rev}}$ is the estimated reversal potential of GABA ($E_{\text{GABA}}$= −36.5 mV)^22^ and g_syn_ is a monoexponential decay (Equation 3).

(2) $$I_{\mathrm{dc}}=g_{\mathrm{syn}}*\left(V_{m}-E_{\mathrm{rev}}\right)$$

(3) $$g_{\mathrm{syn}}={g_{\max}}^{*}\;\exp\left(-t/\mathrm{tau}\right)$$

where *g*_max_ = 1, 2, 5, 10 nS, tau = 7 or 10 ms, and *t* = 0 at the time of the trigger.

The holding current for each recording was adjusted to maintain the membrane potential between −63.5 and −66.5 mV. Pilot studies were conducted to determine the recording sweep duration needed for membrane response to recover and if stimulus order influenced the response. Initial recording sweeps were 5.5 s with the simulated conductances triggered at 0.5 s. These initial recordings showed that the responses of GnRH neurons had returned to baseline within 1 s after the start of the test conductance, thus the duration following initiation of the simulated conductance was decreased to 3 s. Further, using this interval, randomizing the order of the simulated GABAergic conductances did not alter the response of GnRH neurons. For each cell, a series of 10 applications of each conductance was applied in either an ascending or descending order for each time constant.

### 2.8 Data analysis

Simulated GABAergic conductances elicited two types of responses: a subthreshold dynamic‐clamp‐induced postsynaptic potential (dcPSP) or a dcPSP during which an action potential (AP) occurred; the latter category was further subdivided based on timing, see below. To analyze dcPSPs without AP contamination, a minimum of five traces that had stable baselines within the required range (−63.5 to −66.6 mV) for 10 ms before the start of the simulated conductance were averaged. The average trace was analyzed for the following parameters. Latency was calculated as the time from the start of the simulated GABA conductance to the peak of the dcPSP. Amplitude was the difference between the prestimulus baseline and the peak of the dcPSP. The decay time was the time from 80% to 20% of the dcPSP (ms).

To examine the effects of capacitance in isolation on dcPSP shape, an *in silico* RC circuit was used. Resistance was set to the mean input resistance for all cells recorded (737 ± 24 MΩ, 74 cells) and capacitance varied in 2 pF increments from 8 to 16 pF, which spanned over 85% of the values measured in response to the simulated GABAergic conductances tested in the dynamic clamp recordings. The change in voltage over a time step of ∆t is given by Equation (4).

(4) $$∆V=-\frac{∆t}{C}\left(I_{\mathrm{syn}}+I_{\text{leak}}\right)$$

*I*_syn_ is based on Equations (2) and (3), and *I*_leak_ = *g*_leak_(*V* − *E*_leak_). *g*_leak_ is the inverse of the mean input resistance and *E*_leak_ was set to −65 mV. ∆t was set to 0.1 ms.

In dynamic clamp recordings, some GnRH neurons responded to the simulated GABA conductances with a single action potential; most of these arose on the rising phase of the dcPSP, but a subset arose during the decay phase of the dcPSP. Properties of these action potentials were analyzed. Specifically, action potential threshold was defined as the time and potential at which the slope of the second derivative of the membrane potential exceeded 10,000 V/s/s. Action potential latency was the time from the start of the simulated GABA conductance to threshold. Amplitude was calculated as the change in membrane potential from threshold to peak of the action potential. The rate of rise was the maximum voltage derivative from threshold to the peak of the action potential. The full width at half maximum (FWHM) was calculated at the midpoint between threshold and peak. The afterhyperpolarization potential (AHP) time and amplitude were measured relative to threshold.

### 2.9 Statistics

Data were tested for normal distribution with the Shapiro–Wilk test. Data are reported as the mean ± SEM or median ± IQR, as appropriate with the individual values shown when practical. Statistical tests were chosen based on the experimental design and data distribution. Information regarding the statistical tests and data distribution are specified in the results section and tables. Statistical comparisons were made in Prism 10.1.1 (GraphPad Software), except for the statistical analysis of the proportion of time spent in each estrous cycle phase, for which R was used,^30^, ^31^, ^32^, ^33^, ^34^ as Prism is unable to run the appropriate chi‐squared tests. Analysis in R (R version 4.3.1 “Beagle Scouts”) using RStudio^35^ and a combination of open‐sources packages including, rstatix^32^ and flextable,^31^ for which the “chisq_test”, “chisq_descriptives” “row_wise_fishers_test” and the “p.adjust.method” functions were used to run the chi‐squared tests. No statistical analyses were run on the action potential properties measured because of the low number of cells. Significance was set at *p* < 0.05.

## 3 RESULTS

### 3.1 Verification of PNA phenotype

**FIGURE 1:**
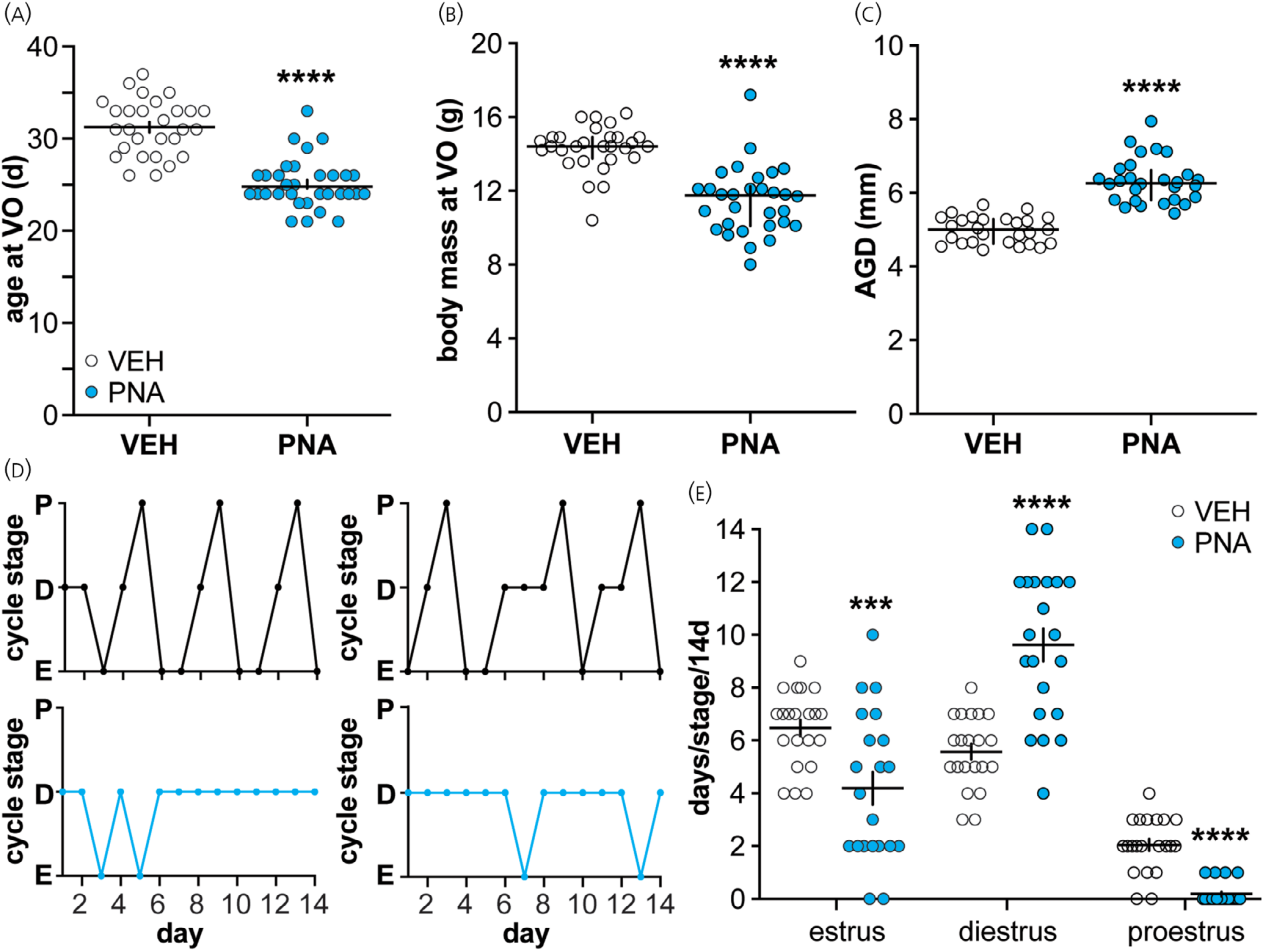
Verification of PNA phenotype. (Individual values and median ± interquartile range for age at vaginal opening (VO, A), body mass at VO (B) and anogenital distance in adulthood (AGD, C). (D) Representative estrous cycles over 14 days beginning postnatal day 70. P, proestrus; D, diestrus; E, estrus (black, vehicle; blue, PNA). (E) Individual values ± SEM for days in each cycle over 14 days. Statistical parameters are in Table 1. ****p < 0.0001.

The reported PNA‐induced differences in phenotype^18^, ^20^, ^21^ were confirmed in this study (Figure 1, Table 1). PNA females were younger at vaginal opening (VO) than VEH females (Figure 1A, Mann–Whitney *U* test, *U* 57.5, *p* < 0.0001). Body mass at VO was lower in PNA than VEH females (Figure 1B, Mann–Whitney *U* test, *p* < 00001) and anogenital distance (AGD) was longer in adult PNA than VEH females (Figure 1C, Mann–Whitney *U* test, *p* < 0.0001). Estrous cycles were disrupted in adult PNA females (Figures 1D, E). Specifically, PNA females spent more time in diestrus (*p* < 0.0001) and less time in estrus (*p* < 0.0002) and proestrus (*p* < 0.0001) than VEH females (*χ* ^2^).

**TABLE 1:** Statistical parameters characterizing the PNA phenotype (Figure 1).

Property	VEH median and interquartile range (IQR)	PNA median and interquartile range (IQR)	Mann–Whitney *U* test	Two‐tailed *p*‐value
Age at vaginal opening (VO)	31.0 (IQR 28.25–33.0)	24.0 (IQR 24.0–26.0)	U = 57.50	**<0.0001**
Body mass at VO (g)	14.9 (IQR 14.4–15.1)	12.1 (IQR 11.1–13.3)	U = 45	**<0.0001**
AGD (mm)	5.11 (IQR 4.63–5.34)	6.33 (IQR 6.16–6.66)	U = 13	**<0.0001**

Mean ± SEM for days spent across estrous cycle stage over 2 weeks		
Cycle stage	VEH	PNA
Estrus	5.9 ± 0.3	3.0 ± 0.6
Diestrus	6.0 ± 0.4	10.8 ± 0.6
Proestrus	2.1 ± 0.2	0.2 ± 0.1

Property	*χ* ^2^ test		
Estrous cycle stage distribution	*χ* ^2^ = 115.57, *n* = 690, df = 2, ***p* < 0.0001**		

	Estrus	Diestrus	Proestrus
Standard residual	6.94	3.17	10.57
Fisher's exact test; Bonferroni adjusted	***p* < 0.0001**	***p* < 0.0001**	***p* < 0.0001**

*Note*: Bold indicates *p* < 0.05.

### 3.2 The decay time constant of GABAergic PSCs in GnRH neurons is increased with age but not affected by PNA treatment

**FIGURE 2:**
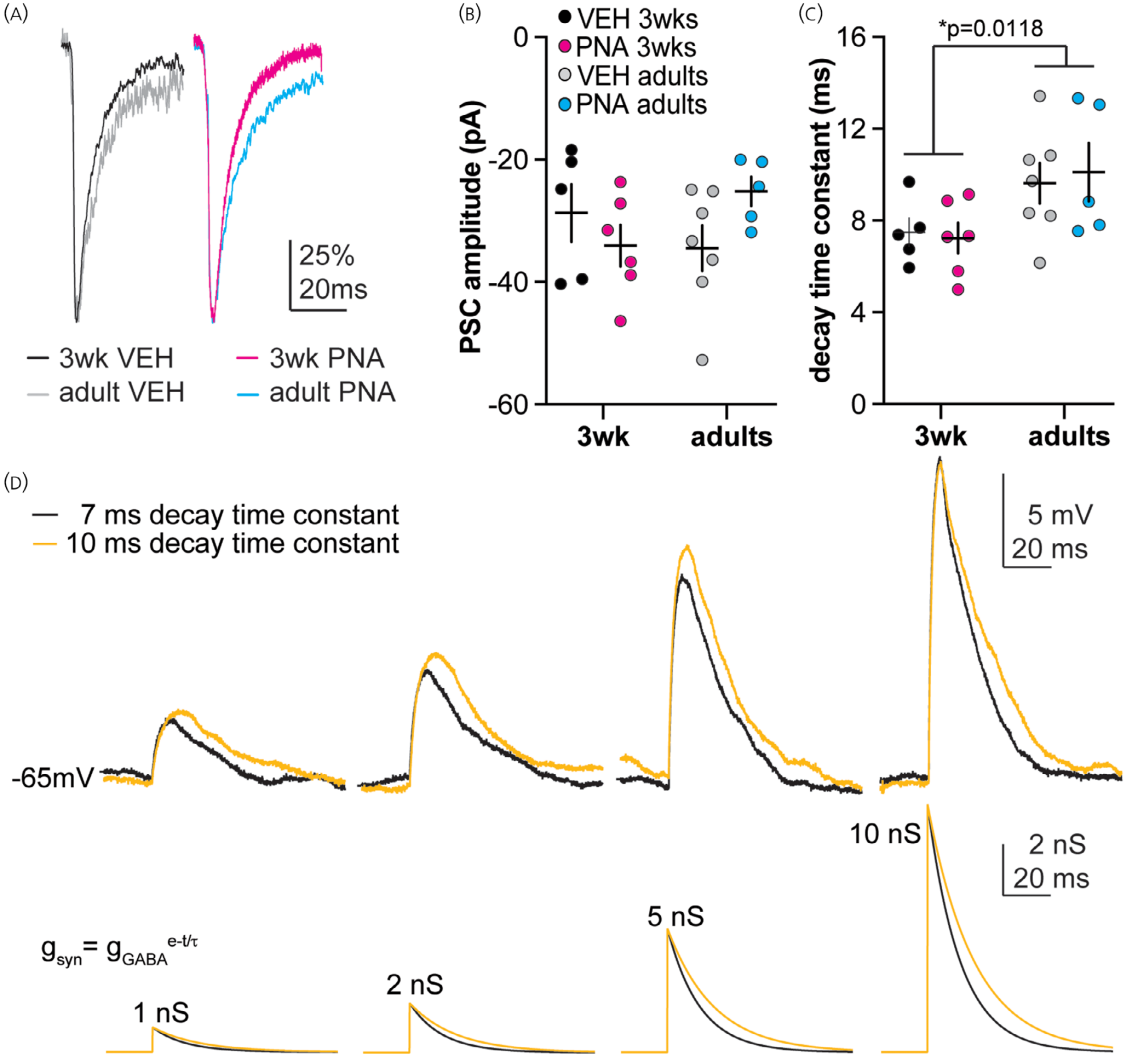
(A) Normalized GABAergic PSCs from each group; VEH on left, PNA on right. (B, C) Individual values and mean ± SEM for PSC amplitude (B) and decay time constant (C). Note 20 mM Cl^−^ in the recording pipette reduces driving force at the holding potential and thus amplitude. (D) GABAergic conductances (bottom, 1, 2, 5, and 10 nS) modeled with a 7 ms (black) or 10 ms (orange) decay time constant and representative examples of dcPSPs in the adult VEH group (top).

Whole‐cell voltage‐clamp recordings were used to measure the properties of isolated GABAergic postsynaptic currents (PSCs) in GnRH neurons from 3‐week‐old and adult VEH and PNA female mice with a physiologic intracellular chloride concentration (3‐week VEH *n* = 5 cells, 3‐week PNA *n* = 6 cells, adult VEH *n* = 6 cells, adult PNA *n* = 5 cells). Figure 2A shows example normalized traces from each group. Neither development nor PNA treatment altered the amplitude of the GABAergic PSCs (Figure 2B, two‐way ANOVA, Table 2). There was, however, a developmental change in the decay kinetics of the GABAergic PSCs (Figure 2C). Specifically, the decay time constant was faster (*p* = 0.0007) in cells from 3‐week‐old VEH (*n* = 5 cells) and PNA females (*n* = 6 cells; 7.4 ± 0.13 ms) than adult VEH (*n* = 6 cells) and PNA females (*n* = 6 cells; 9.9 ± 0.25 ms). Based on these findings, four different test conductances (1, 2, 5 and 10 nS) with a reversal potential of −36.5 mV^22^ were delivered. Each GABA conductance was simulated twice using a monoexponential decay time constant of 7 ms or 10 ms, for a total of eight different dynamic‐clamp conditions. Representative examples of the simulated GABA conductances and resulting dcPSPs are in Figure 2D for the adult VEH group.

**TABLE 2:** Descriptive statistics and statistical parameters from two‐way ANOVA for GABAergic post‐synaptic currents (Figure 2).

Property	Age	Treatment		Interaction	
Mean PSC amplitude (pA)	Diff, −1.56 [Cl. −9.33, 6.21] *F*(1,19) = 0.18, *p* = 0.679	Diff, −1.96 [Cl. −9.73, 5.81] *F*(1,19) = 0.28, *p* = 0.604		Diff, 14.65 [Cl. −0.90, 30.19] *F*(1,19) = 3.89, *p* = 0.063	
Decay time constant (ms)	Diff, −2.50 [Cl. −4.38, −0.623] *F*(1,19) = 7.76, ***p* = 0.011**	Diff, −0.1179 [Cl. −1.99, 1.76] *F*(1,19) = 0.01723, *p* = 0.896		Diff, 0.75 [Cl. −3.01, 4.51] *F*(1,19) = 0.18, *p* = 0.680	
	*Šídák's* multiple comparisons	3‐week VEH versus 3‐week PNA	VEH adult versus PNA adult	3‐week VEH versus VEH adult	3‐week PNA versus PNA adult
		*p* = 0.999	*p* = 0.991	*p* = 0.358	*p* = 0.143

*Note*: Bold indicates *p* < 0.05.

### 3.3 Kinetic properties of dynamic‐clamp‐induced postsynaptic potentials (dcPSPs) vary largely in a manner consistent with changes in passive membrane properties

**FIGURE 3:**
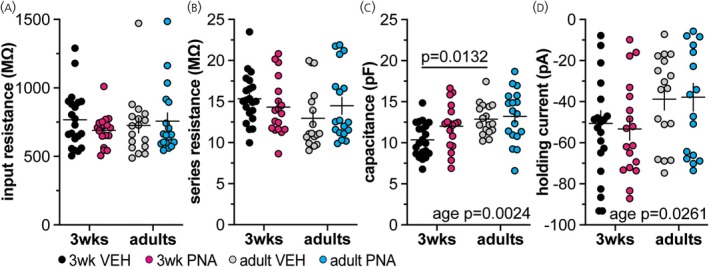
Recording quality and passive properties of GnRH neurons. (A–D) Individual values and mean ± SEM for series resistance (A), input resistance (B), capacitance (C), and holding current (D). Two‐way ANOVA parameters are in Table 3.

There were no differences in input resistance (Figure 3A) or series resistance (Figure 3B) among groups of GnRH neurons in the dynamic clamp studies. There was, however, an age‐induced increase in the capacitance (Figure 3C). Less hyperpolarizing current was required to maintain GnRH neurons from adults between −63.5 mV and −66.5 mV than those from 3‐week‐old mice (two‐way ANOVA, Figure 3D; Table 3). There was no difference in baseline membrane potential at the time of application of the simulated GABA conductance other than a mild interaction between age and treatment for the 1 nS/7 ms decay time conductance groups (Table 4); this did not contribute to any differences among these groups in dcPSP properties below.

**TABLE 3:** Descriptive statistics (mean ± SEM) and statistical analysis of dynamic clamp recording quality parameters (Figure 3).

Property (mean ± SEM)	3‐week VEH (*n* = 12)	3‐week PNA (*n* = 13)	Adult VEH (*n* = 14)	Adult PNA (*n* = 12)
Input resistance (MΩ)	767.905 ± 45.54	690.51 ± 27.02	726.09 ± 55.24	757.91 ± 59.37
Series resistance (MΩ)	15.33 ± 0.66	14.32 ± 0.81	12.96 ± 0.91	14.48 ± 1.02
Capacitance (pF)	10.37 ± 0.45	12.02 ± 0.65	12.88 ± 0.47	13.23 ± 0.76
Holding current (pA)	−50.58 ± 6.15	−53.29 ± 5.44	−38.81 ± 5.25	−37.83 ± 6.74

Two‐way ANOVA (passive properties and recording quality parameters of dynamic clamp recordings)					
Property	Age	Treatment		Interaction	
Input resistance (MΩ)	Diff, 48.30 [Cl, −109.1, 83.54] *F*(1,70) = 0.0701; *p* = 0.7919	Diff, 22.79 [Cl, −73.55, 119.1] *F*(1,70) = 0.2226; *p* = 0.6385		Diff, 109.2 [Cl, −83.46, 301.9] *F*(1,70) = 1.278; *p* = 0.2621	
Series resistance (MΩ)	Diff, 1.109 [Cl, −0.5853, 2.804] *F*(1,70) = 1.705; *p* = 0.1960	Diff, −0.2575 [Cl, −1.952, 1.437] *F*(1,70) = 0.0918; *p* = 0.7627		Diff, 2.537 [Cl, −0.852, 5.926] *F*(1,70) = 2.229; *p* = 0.1399	
Capacitance (pF)	Diff, −1.865 [Cl, −3.044, 0.6856] *F*(1,70) = 9.950; ***p* = 0.0024**	Diff, 0.5911 [Cl, −2.177, 0.181] *F*(1,70) = 2.850; *p* = 0.0958		Diff, −1.301 [Cl, −3.659, 1.057] *F*(1,70) = 1.211; *p* = 0.2750	
	*Šídák's* multiple comparisons	VEH 3 weeks versus VEH adults	PNA 3 weeks versus PNA adults	VEH 3 weeks versus PNA 3 weeks	VEH adults versus PNA adults
		***p* = 0.0132**	*p* = 0.4905	*p* = 0.1742	*p* = 0.9903
Holding current (pA)	Diff, 0.8676 [Cl, −11.08, 12.82] *F*(1,70) = 0.0201; *p* = 0.8852	Diff, −13.62 [Cl, −25.57, −1.672] *F*(1,70) = 5.171; ***p* = 0.0261**		Diff, 3.689 [Cl, −20.21, 27.58] *F*(1,70) = 0.0948; *p* = 0.7590	
	*Šídák's* multiple comparisons	VEH 3 weeks versus VEH adults	PNA 3 weeks versus PNA adults	VEH 3 weeks versus PNA 3 weeks	VEH adults versus PNA adults
		*p* = 5052	*p* = 0.2749	*p* = 0.9955	*p* > 0.9999

Property (mean ± SEM)	3‐week VEH (*n* = 12)	3‐Week PNA (*n* = 13)	Adult VEH (*n* = 14)	Adult PNA (*n* = 12)
Input resistance (MΩ)	767.905 ± 45.54	690.51 ± 27.02	726.09 ± 55.24	757.91 ± 59.37
Series resistance (MΩ)	15.33 ± 0.66	14.32 ± 0.81	12.96 ± 0.91	14.48 ± 1.02
Capacitance (pF)	10.37 ± 0.45	12.02 ± 0.65	12.88 ± 0.47	13.23 ± 0.76
Holding current (pA)	−50.58 ± 6.15	−53.29 ± 5.44	−38.81 ± 5.25	−37.83 ± 6.74

Two‐way ANOVA (passive properties and recording quality parameters of dynamic clamp recordings)					
Property	Age	Treatment		Interaction	
Input resistance (MΩ)	Diff, 48.30 [Cl, −109.1, 83.54] *F*(1,70) = 0.0701; *p* = 0.7919	Diff, 22.79 [Cl, −73.55, 119.1] *F*(1,70) = 0.2226; *p* = 0.6385		Diff, 109.2 [Cl, −83.46, 301.9] *F*(1,70) = 1.278; *p* = 0.2621	
Series resistance (MΩ)	Diff, 1.109 [Cl, −0.5853, 2.804] *F*(1,70) = 1.705; *p* = 0.1960	Diff, −0.2575 [Cl, −1.952, 1.437] *F*(1,70) = 0.0918; *p* = 0.7627		Diff, 2.537 [Cl, −0.852, 5.926] *F*(1,70) = 2.229; *p* = 0.1399	
Capacitance (pF)	Diff, −1.865 [Cl, −3.044, 0.6856] *F*(1,70) = 9.950; ***p* = 0.0024**	Diff, 0.5911 [Cl, −2.177, 0.181] *F*(1,70) = 2.850; *p* = 0.0958		Diff, −1.301 [Cl, −3.659, 1.057] *F*(1,70) = 1.211; *p* = 0.2750	
	*Šídák's* multiple comparisons	VEH 3 weeks versus VEH adults	PNA 3 weeks versus PNA adults	VEH 3 weeks versus PNA 3 weeks	VEH adults versus PNA adults
		***p* = 0.0132**	*p* = 0.4905	*p* = 0.1742	*p* = 0.9903
Holding current (pA)	Diff, 0.8676 [Cl, −11.08, 12.82] *F*(1,70) = 0.0201; *p* = 0.8852	Diff, −13.62 [Cl, −25.57, −1.672] *F*(1,70) = 5.171; ***p* = 0.0261**		Diff, 3.689 [Cl, −20.21, 27.58] *F*(1,70) = 0.0948; *p* = 0.7590	
	*Šídák's* multiple comparisons	VEH 3 weeks versus VEH adults	PNA 3 weeks versus PNA adults	VEH 3 weeks versus PNA 3 weeks	VEH adults versus PNA adults
		*p* = 5052	*p* = 0.2749	*p* = 0.9955	*p* > 0.9999

*Note*: Bold indicates *p* < 0.05.

**TABLE 4:** Statistical parameters from two‐way ANOVA for the initial baseline membrane of GnRH neurons.

Baseline Membrane Potential (mV) two‐way ANOVA					
	Age	Treatment		Interaction	
dcGABA conductances (7 ms decay time constant)					
1 nS	Diff, −0.0695 [Cl, −0.3965, 0.2575] *F*(1,44) = 0.184; *p* = 0.6705	Diff, −0.2063 [Cl, −0.5333, 0.1207] *F*(1,44) = 1.616; *p* = 0.2103		Diff, −0.6819 [Cl, −1.336, −0.0279] *F*(1,44) = 4.416; ***p* = 0.0414**	
	*Šídák's* multiple comparisons	VEH 3 weeks versus VEH adults	PNA 3 weeks versus PNA adults	VEH 3 weeks versus PNA 3 weeks	VEH adults versus PNA adults
		*p* = 0.2661	*p* = 0.6882	*p* = 0.0929	*p* = 0.9597
2 nS	Diff, −0.0198 [Cl, −0.2946, 0.2551] *F*(1,46) = 0.0201; *p* = 0.8856	Diff, 0.1764 [Cl, −0.0984, 0.4512] *F*(1,46) = 1.669; *p* = 0.2028		Diff, −0.3805 [Cl, −0.9301, 0.1692] *F*(1,46) = 1.941; *p* = 0.1702	
5 nS	Diff, −0.1373 [Cl, −0.4804, 0.2059] *F*(1,49) = 0.6462; *p* = 0.4254	Diff, 0.1649 [Cl, −0.1782, 0.5080] *F*(1,49) = 0.9327; *p* = 0.3389		Diff, 0.0870 [Cl, −0.5992, 0.7732] *F*(1,49) = 0.0649; *p* = 0.7999	
10 nS	Diff, −0.3378 [Cl, −0.6843, 0.0087] *F*(1,47) = 3.847; *p* = 0.0558	Diff, −0.1432 [Cl, −0.2033, 0.4897] *F*(1,47) = 0.6912; *p* = 0.4100		Diff, −0.1326 [Cl, −0.8256, 0.5604] *F*(1,47) = 0.1482; *p* = 0.7020	
dcGABA conductances (10 ms decay time constant)					
1 nS	Diff, 0.1990 [Cl, −0.1602, 0.5582] *F*(1,41) = 1.252; *p* = 0.2697	Diff, 0.2136 [Cl, −0.1456, 0.5728] *F*(1,41) = 1.442; *p* = 0.2367		Diff, −0.6113 [Cl, −1.330, 0.1071] *F*(1,41) = 2.953; *p* = 0.0932	
2 nS	Diff, −0.0058 [Cl, −0.4171, 0.4054] *F*(1,42) = 0.0008; *p* = 0.9773	Diff, 0.2911 [Cl, −0.1202, 0.7023] *F*(1,42) = 2.040; *p* = 0.1606		Diff, −0.2827 [Cl, −1.105, 0.5398] *F*(1,42) = 0.4811; *p* = 0.4918	
5 nS	Diff, −0.1601 [Cl, −0.5168, 0.1967] *F*(1,47) = 0.8148; *p* = 0.3713	Diff, 0.2349 [Cl, −0.1218, 0.5917] *F*(1,47) = 1.755; *p* = 0.1916		Diff, −0.4668 [Cl, −1.180, 0.2467] *F*(1,47) = 1.732; *p* = 0.1945	
10 nS	Diff, −0.2543 [Cl, −0.6099, 0.1014] *F*(1,44) = 2.076; *p* = 0.1567	Diff, −0.0137 [Cl, −0.3687, 0.3426] *F*(1,44) = 0.0054; *p* = 0.9413		Diff, −0.5883 [Cl, −1.300, 0.1230] *F*(1,44) = 2.778; *p* = 0.1027	

*Note*: Bold indicates *p* < 0.05.

**FIGURE 4:**
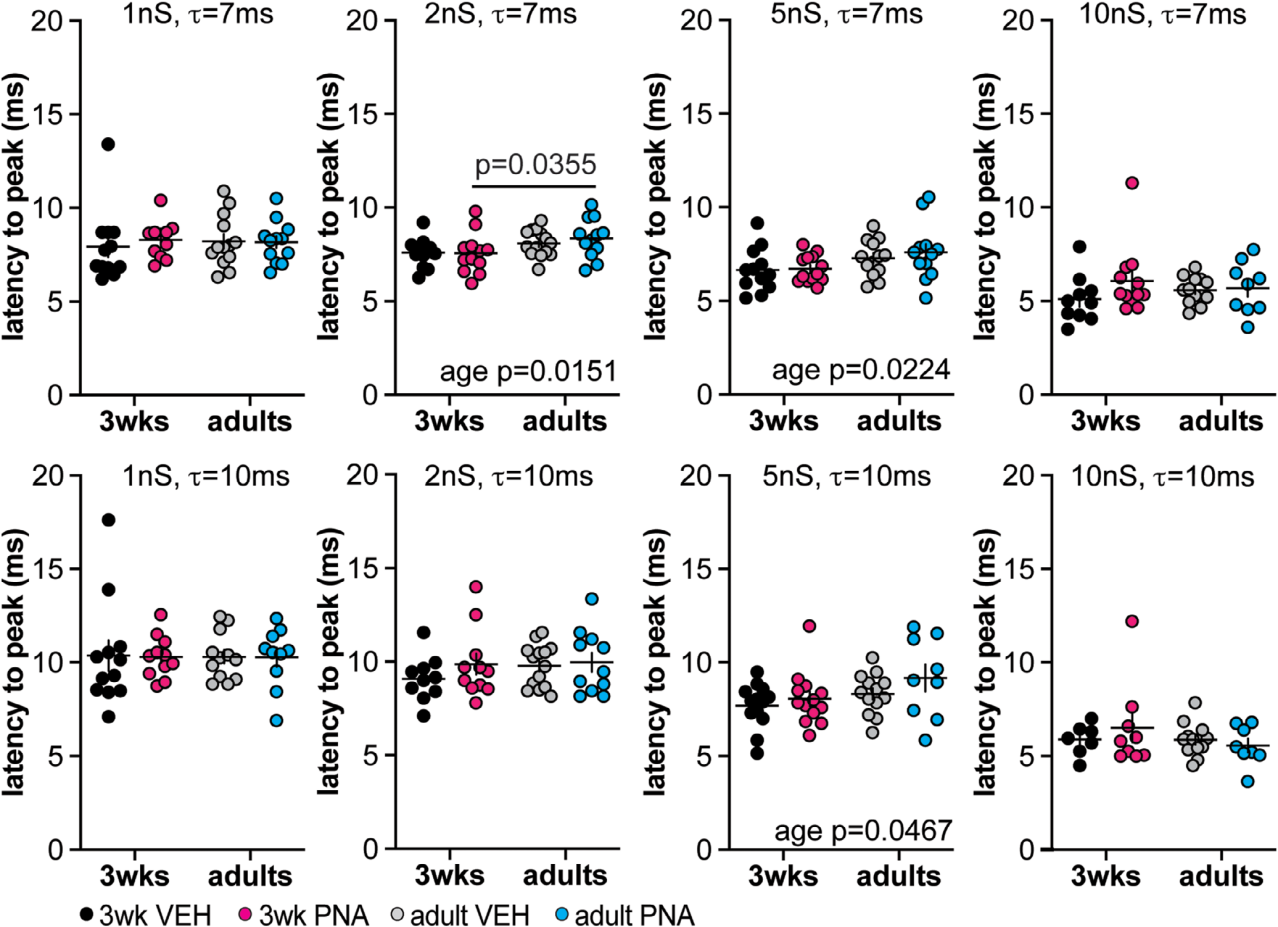
Latency to GnRH neuron dc PSP peak. Individual values ± SEM for the latency (ms) to reach peak subthreshold membrane response. Modeled conductances with a 7 ms decay time constant (*τ*) are in the top row and those with a 10 ms decay time constant are in the bottom row. Two‐way ANOVA and *Šídák's* multiple comparisons test parameters are in Table 8.

**FIGURE 5:**
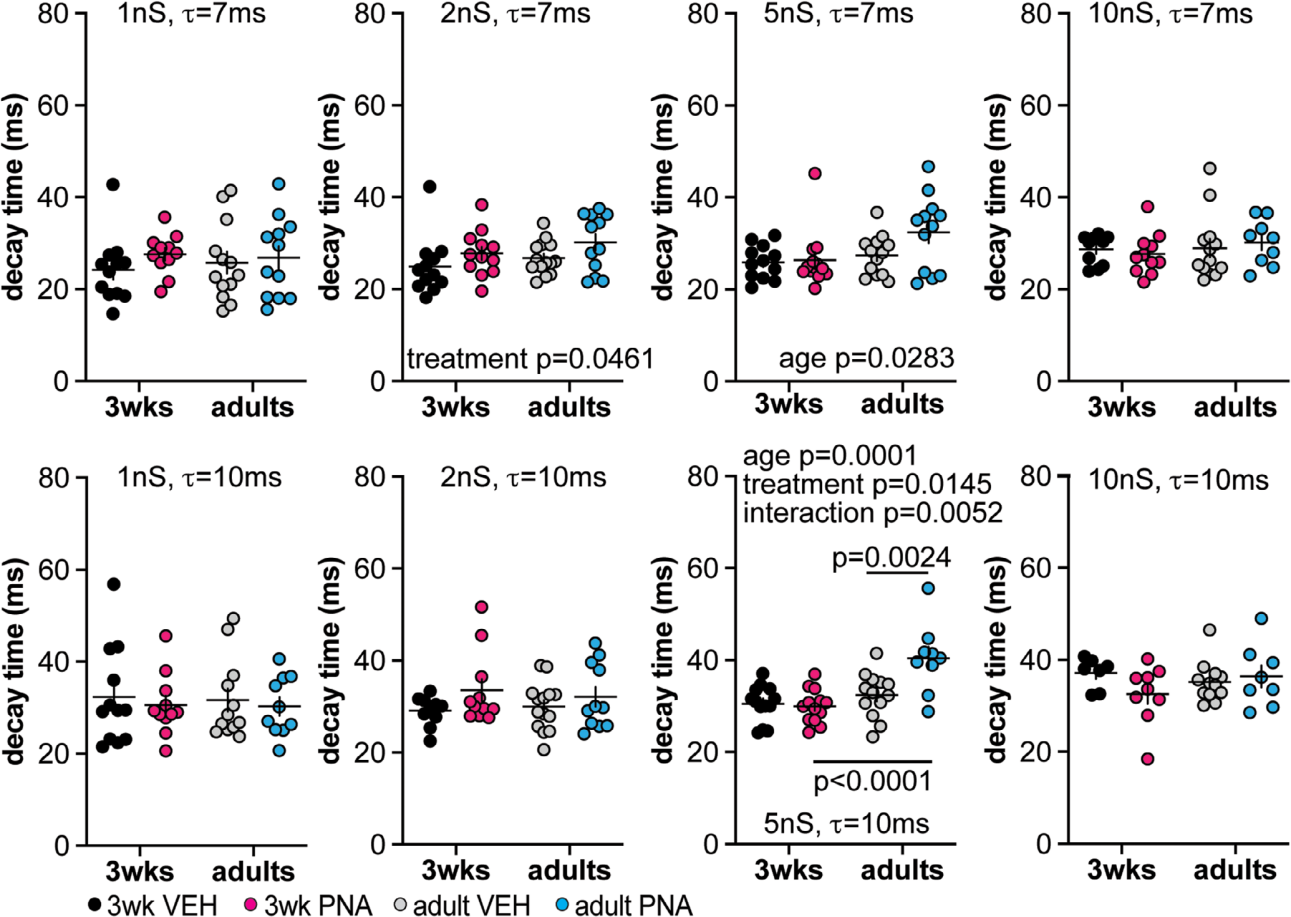
The 80/20 decay time of GnRH neuron dcPSPs. Individual values ± SEM for the time it takes GnRH neurons to repolarize following the peak subthreshold membrane response (calculated from 80% to 20% of the peak). Modeled conductances with a 7 ms decay time constant (*τ*) are in the top row and those with a 10 ms decay time constant are in the bottom row. Two‐way ANOVA and *Šídák's* multiple comparisons parameters are in Table 7.

Simulated GABAergic conductances (1, 2, 5 or 10 nS) with decay time constants of 7 or 10 ms were applied to GnRH neurons from the four groups. Table 5 shows the total number of cells included for each group. There were mild development‐induced increases in the latency to peak (Figure 4, Table 6) observed for the middle amplitude conductances (2 and 5 nS) with a 7 ms decay time constant; these shifts were also evident for the 5 nS/10 ms conductance. These developmental shifts may be attributable to the developmental increase in capacitance. A similar pattern of differences was observed regarding an increase in the 80/20 decay time in adults but with the addition of an age by PNA treatment interaction and effect of treatment increasing decay time in cells from adult PNA mice (Figure 5, Table 7). The influence of PNA treatment suggests a shift in the population of voltage‐gated channels that are engaged by the depolarization of the dcPSP, with the channels in adult PNA mice acting to prolong the depolarization.

**TABLE 5:** Number of cells and number of mice included in each protocol and membrane response in terms of postsynaptic potentials only (PSPs), action potentials (APs), or both.

Group	# cells and # of mice by conductance (nS)/decay time constant (ms)							
	1 nS/7 ms	1 nS/10 ms	2 nS/7 ms	2 nS/10 ms	5 nS/7 ms	5 ns/10 ms	10 nS/7 ms	10 ns/10 ms
3‐week VEH								
PSP	# cells = 12	# cells = 12	# cells = 12	# cells = 10	# cells = 10	# cells = 9	# cells = 10	# cells = 7
	# mice = 8	# mice = 8	# mice = 8	# mice = 8	# mice = 5	# mice = 5	# mice = 4	# mice = 4
AP	# cells = 0	# cells = 0	# cells = 0	# cells = 0	# cells = 3	# cells = 2	# cells = 4	# cells = 7
	# mice = 0	# mice = 0	# mice = 0	# mice = 0	# mice = 3	# mice = 2	# mice = 4	# mice = 6
Both	# cells = 0	# cells = 0	# cells = 0	# cells = 0	# cells = 2	# cells = 4	# cells = 1	# cells = 0
	# mice = 0	# mice = 0	# mice = 0	# mice = 0	# mice = 2	# mice = 4	# mice = 1	# mice = 0
3‐week PNA								
PSP	# cells = 11	# cells = 11	# cells = 11	# cells = 11	# cells = 11	# cells = 13	# cells = 10	# cells = 9
	# mice = 9	# mice = 8	# mice = 8	# mice = 8	# mice =10	# mice = 10	# mice = 5	# mice = 6
AP	# cells = 0	# cells = 0	# cells = 0	# cells = 0	# cells = 0	# cells = 0	# cells = 2	# cells = 1
	# mice = 0	# mice = 0	# mice = 0	# mice = 0	# mice = 0	# mice = 0	# mice = 2	# mice = 1
Both	# cells = 0	# cells = 0	# cells = 0	# cells = 0	# cells = 0	# cells = 0	# cells = 2	# cells = 3
	# mice = 0	# mice = 0	# mice = 0	# mice = 0	# mice = 0	# mice = 0	# mice = 2	# mice = 2
Adult VEH								
PSP	# cells = 13	# cells = 12	# cells = 12	# cells = 14	# cells = 14	# cells = 12	# cells = 11	# cells = 12
	# mice = 7	# mice = 8	# mice = 8	# mice = 8	# mice = 8	# mice = 6	# mice = 5	# mice = 6
AP	# cells = 0	# cells = 0	# cells = 0	# cells = 0	# cells = 0	# cells = 0	# cells = 0	# cells = 0
	# mice = 0	# mice = 0	# mice = 0	# mice = 0	# mice = 0	# mice = 0	# mice = 0	# mice = 0
Both	# cells = 0	# cells = 0	# cells = 0	# cells = 0	# cells = 0	# cells = 1	# cells = 2	# cells = 1
	# mice = 0	# mice = 0	# mice = 0	# mice = 0	# mice = 0	# mice = 1	# mice = 2	# mice = 1
Adult PNA								
PSP	# cells = 12	# cells = 12	# cells = 10	# cells = 12	# cells = 12	# cells = 8	# cells = 8	# cells = 8
	# mice = 5	# mice = 10	# mice = 10	# mice = 10	# mice = 10	# mice = 7	# mice = 5	# mice = 6
AP	# cells = 0	# cells = 0	# cells = 0	# cells = 0	# cells = 0	# cells = 1	# cells = 2	# cells = 4
	# mice = 0	# mice = 0	# mice = 0	# mice = 0	# mice = 0	# mice = 1	# mice = 2	# mice = 2
Both	# cells = 0	# cells = 0	# cells = 0	# cells = 0	# cells = 0	# cells = 1	# cells = 2	# cells = 2
	# mice = 0	# mice = 0	# mice = 0	# mice = 0	# mice = 0	# mice = 1	# mice = 2	# mice = 2

**TABLE 6:** Statistical parameters from two‐way ANOVA for the latency in milliseconds from the start of the dcGABA conductance to the peak dcPSP amplitude (Figure 4).

Latency (ms; two‐way ANOVA)					
	Age	Treatment		Interaction	
dcGABA conductances (7 ms decay time constants)					
1 nS	Diff, −0.0789 [Cl, −0.9046, 0.7468] *F*(1,44) = 0.0371; *p* = 0.8482	Diff, −0.1544 [Cl, −0.9801, 0.6712] *F*(1,44) = 0.1421; *p* = 0.7080		Diff, −0.3980 [Cl, −2.049, 1.253] *F*(1,44) = 0.2359; *p* = 0.6296	
2 nS	Diff, −0.119 [Cl, −0.6308, 0.4070] *F*(1,46) = 0.1885; *p* = 0.6662	Diff, −0.119 [Cl, −0.6308, 0.4070] *F*(1,46) = 0.1885; *p* = 0.6662		Diff, 0.3071 [Cl, −0.7306, 1.345] *F*(1,46) = 0.3549; *p* = 0.5543	
	*Šídák's* multiple comparisons	VEH 3 weeks versus VEH adults	PNA 3 weeks versus PNA adults	VEH 3 weeks versus PNA 3 weeks	VEH adults versus PNA adults
		*p* = 0.1714	***p* = 0.0355**	*p* = 0.9111	*p* = 0,4618
5 nS	Diff, −0.7516 [Cl, −1.392, −0.1115] *F*(1,46) = 5.586; *p* = **0.0224**	Diff, −0.1901 [Cl, −0.8302, 0.4501] *F*(1,46) = 0.3572; *p* = 0.5530		Diff, −0.2654 [Cl, −1.015, 1.546] *F*(1,46) = 0.1741; *p* = 0.6784	
	*Šídák's* multiple comparisons	VEH 3 weeks versus VEH adults	PNA 3 weeks versus PNA adults	VEH 3 weeks versus PNA 3 weeks	VEH adults versus PNA adults
		*p* = 0.5377	*p* = 0.2036	*p* = 0.9999	*p* = 0.9250
10 nS	Diff, −0.0433 [Cl, −0.8664, 0.7798] *F*(1,40) = 0.0113; *p* = 0.9158	Diff, −0.5456 [Cl, −1.369, 0.2775] *F*(1,40) = 1.795; *p* = 0.1879		Diff, −0.8672 [Cl, −2.513, 0.7789] *F*(1,40) = 1.134; *p* = 0.2934	
dcGABA conductances (10 ms decay time constants)					
1 nS	Diff, 0.0362 [Cl, −1.093, 1.165] *F*(1,41) = 0.0042; *p* = 0.9487	Diff, 0.0487 [Cl, −1.080, 1.178] *F*(1,41) = 0.0076; *p* = 0.9310		Diff, 0.,0324 [Cl, −2.225, 2.290] *F*(1,41) = 0.0008; *p* = 0.9770	
2 nS	Diff, −0.4029 [Cl, −1.293, 0.4867] *F*(1,42) = 0.8353; *p* = 0.3660	Diff, −0.4798 [Cl, −1.369, 0.4098] *F*(1,42) = 1.185; *p* = 0.2826		Diff, −0.5785 [Cl, −2.358, 1.201] *F*(1,42) = 0.4305; *p* = 0.5153	
5 nS	Diff, −0.8697 [Cl, −1.726, −0.0131] *F*(1,44) = 4.187; ***p* = 0.0476**	Diff, −0.6043 [Cl, −1.461, 0.2523] *F*(1,44) = 2.021; *p* = 0.1621		Diff, 0.4932 [Cl, −1.220, 2.206] *F*(1,44) = 0.3366; *p* = 0.5648	
	*Šídák's* multiple comparisons	VEH 3 weeks versus VEH adults	PNA 3 weeks versus PNA adults	VEH 3 weeks versus PNA 3 weeks	VEH adults versus PNA adults
		*p* = 0.7320	*p* = 0.2946	*p* = 0.9527	*p* = 0.5567
10 nS	Diff, 0.4725 [Cl, −0.5027, 1.448] *F*(1,32) = 0.9739; *p* = 0.3311	Diff, −0.1564 [Cl, −1.132, 0.8188] *F*(1,32) = 0.1067; *p* = 0.7460		Diff, −0.9378 [Cl, −2.888, 1.013] *F*(1,32) = 0.9593; *p* = 0.3347	

Latency (ms; two‐way ANOVA)					
	Age	Treatment		Interaction	
dcGABA conductances (7 ms decay time constants)					
1 nS	Diff, −0.0789 [Cl, −0.9046, 0.7468] *F*(1,44) = 0.0371; *p* = 0.8482	Diff, −0.1544 [Cl, −0.9801, 0.6712] *F*(1,44) = 0.1421; *p* = 0.7080		Diff, −0.3980 [Cl, −2.049, 1.253] *F*(1,44) = 0.2359; *p* = 0.6296	
2 nS	Diff, −0.119 [Cl, −0.6308, 0.4070] *F*(1,46) = 0.1885; *p* = 0.6662	Diff, −0.119 [Cl, −0.6308, 0.4070] *F*(1,46) = 0.1885; *p* = 0.6662		Diff, 0.3071 [Cl, −0.7306, 1.345] *F*(1,46) = 0.3549; *p* = 0.5543	
	*Šídák's* multiple comparisons	VEH 3 weeks versus VEH adults	PNA 3 weeks versus PNA adults	VEH 3 weeks versus PNA 3 weeks	VEH adults versus PNA adults
		*p* = 0.1714	***p* = 0.0355**	*p* = 0.9111	*p* = 0,4618
5 nS	Diff, −0.7516 [Cl, −1.392, −0.1115] *F*(1,46) = 5.586; *p* = **0.0224**	Diff, −0.1901 [Cl, −0.8302, 0.4501] *F*(1,46) = 0.3572; *p* = 0.5530		Diff, −0.2654 [Cl, −1.015, 1.546] *F*(1,46) = 0.1741; *p* = 0.6784	
	*Šídák's* multiple comparisons	VEH 3 weeks versus VEH adults	PNA 3 weeks versus PNA adults	VEH 3 weeks versus PNA 3 weeks	VEH adults versus PNA adults
		*p* = 0.5377	*p* = 0.2036	*p* = 0.9999	*p* = 0.9250
10 nS	Diff, −0.0433 [Cl, −0.8664, 0.7798] *F*(1,40) = 0.0113; *p* = 0.9158	Diff, −0.5456 [Cl, −1.369, 0.2775] *F*(1,40) = 1.795; *p* = 0.1879		Diff, −0.8672 [Cl, −2.513, 0.7789] *F*(1,40) = 1.134; *p* = 0.2934	
dcGABA conductances (10 ms decay time constants)					
1 nS	Diff, 0.0362 [Cl, −1.093, 1.165] *F*(1,41) = 0.0042; *p* = 0.9487	Diff, 0.0487 [Cl, −1.080, 1.178] *F*(1,41) = 0.0076; *p* = 0.9310		Diff, 0.,0324 [Cl, −2.225, 2.290] *F*(1,41) = 0.0008; *p* = 0.9770	
2 nS	Diff, −0.4029 [Cl, −1.293, 0.4867] *F*(1,42) = 0.8353; *p* = 0.3660	Diff, −0.4798 [Cl, −1.369, 0.4098] *F*(1,42) = 1.185; *p* = 0.2826		Diff, −0.5785 [Cl, −2.358, 1.201] *F*(1,42) = 0.4305; *p* = 0.5153	
5 nS	Diff, −0.8697 [Cl, −1.726, −0.0131] *F*(1,44) = 4.187; ***p* = 0.0476**	Diff, −0.6043 [Cl, −1.461, 0.2523] *F*(1,44) = 2.021; *p* = 0.1621		Diff, 0.4932 [Cl, −1.220, 2.206] *F*(1,44) = 0.3366; *p* = 0.5648	
	*Šídák's* multiple comparisons	VEH 3 weeks versus VEH adults	PNA 3 weeks versus PNA adults	VEH 3 weeks versus PNA 3 weeks	VEH adults versus PNA adults
		*p* = 0.7320	*p* = 0.2946	*p* = 0.9527	*p* = 0.5567
10 nS	Diff, 0.4725 [Cl, −0.5027, 1.448] *F*(1,32) = 0.9739; *p* = 0.3311	Diff, −0.1564 [Cl, −1.132, 0.8188] *F*(1,32) = 0.1067; *p* = 0.7460		Diff, −0.9378 [Cl, −2.888, 1.013] *F*(1,32) = 0.9593; *p* = 0.3347	

*Note*: Bold indicates *p* < 0.05.

**TABLE 7:** Statistical parameters from two‐way ANOVA for the decay time of dcPSPs (Figure 5).

Decay time (ms; two‐way ANOVA)					
	Age	Treatment		Interaction	
dcGABA conductances (7 ms decay time constants)					
1 nS	Diff, −0.4024 [Cl, −4.756, 3.952] *F*(1,44) = 0.035; *p* = 0.8531	Diff, −2.241 [Cl, −6.595, 2.113] *F*(1,44) = 1.076; *p* = 0.3052		Diff, −2.360 [Cl, −11.07, 6.348] *F*(1,44) = 0.2983; *p* = 0.5877	
2 nS	Diff, −2.094 [Cl, −5.131, 0.9433] *F*(1,46) = 1.926; *p* = 0.1719	Diff, −3.093 [Cl, −6.130, −0.0558] *F*(1,46) = 4.202; ***p* = 0.0461**		Diff, 0.5849 [Cl, −5.489, 6.659] *F*(1,46) = 0.038; *p* = 0.8472	
	*Šídák's* multiple comparisons	VEH 3 weeks versus VEH adults	PNA 3 weeks versus PNA adults	VEH 3 weeks versus PNA 3 weeks	VEH adults versus PNA adults
		*p* = 0.8652	*p* = 0.7280	*p* = 0.5984	*p* = 0.3803
5 nS	Diff, −3.730 [Cl, −7.047, 0.4135] *F*(1,46) = 5.125; ***p* = 0.0283**	Diff, −2.740 [Cl, −6.057, 0.5762] *F*(1,46) = 2.766; *p* = 0.1031		Diff, 4.547 [Cl, −2.087, 11.18] *F*(1,46) = 1.904; *p* = 0.1743	
	*Šídák's* multiple comparisons	VEH 3 weeks versus VEH adults	PNA 3 weeks versus PNA adults	VEH 3 weeks versus PNA 3 weeks	VEH adults versus PNA adults
		*p* = 0.9532	*p* = 0.0520	*p* = 0.9994	*p* = 0.1389
10 nS	Diff, −1.420 [Cl, −4.748, 1.908] *F*(1,39) = 0.7445; *p* = 0.3935	Diff, −0.0824 [Cl, −3.410, 3.246] *F*(1,39) = 0.003; *p* = 0.9603		Diff, 2.204 [Cl, −4.452, 8.860] *F*(1,39) = 0.4485; *p* = 0.5070	
dcGABA conductances (10 ms decay time constants)					
1 nS	Diff, 0.444 [Cl, −4.655, 5.543] *F*(1,41) = 0.031; *p* = 0.8612	Diff, 1.545 [Cl, −3.554, 6.644] *F*(1,41) = 0.374; *p* = 0.544		Diff, 0.3803 [Cl, −9.818, 10.58] *F*(1,41) = 0.006; *p* = 0.9403	
2 nS	Diff, 0.253 [Cl, −3.428, 3.934] *F*(1,42) = 0.019; *p* = 0.8903	Diff, −3.320 [Cl, −7.001, 0.3614] *F*(1,42) = 3.312; *p* = 0.0759		Diff, −2.354 [Cl, −9.716, 5.008] *F*(1,42) = 0.4165; *p* = 0.5222	
5 nS	Diff, −6.181 [Cl, −9.113, −3.249] *F*(1,44) = 18.05; ***p* = 0.0001**	Diff, −3.702 [Cl, −6.634, −0.7701] *F*(1,44) = 6.476; ***p* = 0.0145**		Diff, 8.565 [Cl, 2.701, −3.249] *F*(1,44) = 8.666; ***p* = 0.0052**	
	*Šídák's* multiple comparisons	VEH 3 weeks versus VEH adults	PNA 3 weeks versus PNA adults	VEH 3 weeks versus PNA 3 weeks	VEH adults versus PNA adults
		*p* = 0.8056	***p* < 0.0001**	*p* = 0.9971	***p* = 0.002**
10 nS	Diff, −0.939 [Cl, −4.651, 2.771] *F*(1,32) = 0.266; *p* = 0.6095	Diff, 1.684 [Cl, −2.027, 5.395] *F*(1,32) = 0.854; *p* = 0.3622		Diff, 5.747 [Cl, −1.676, 13.17] *F*(1,32) = 2.487; *p* = 0.1246	

*Note*: Bold indicates *p* < 0.05.

**FIGURE 6:**
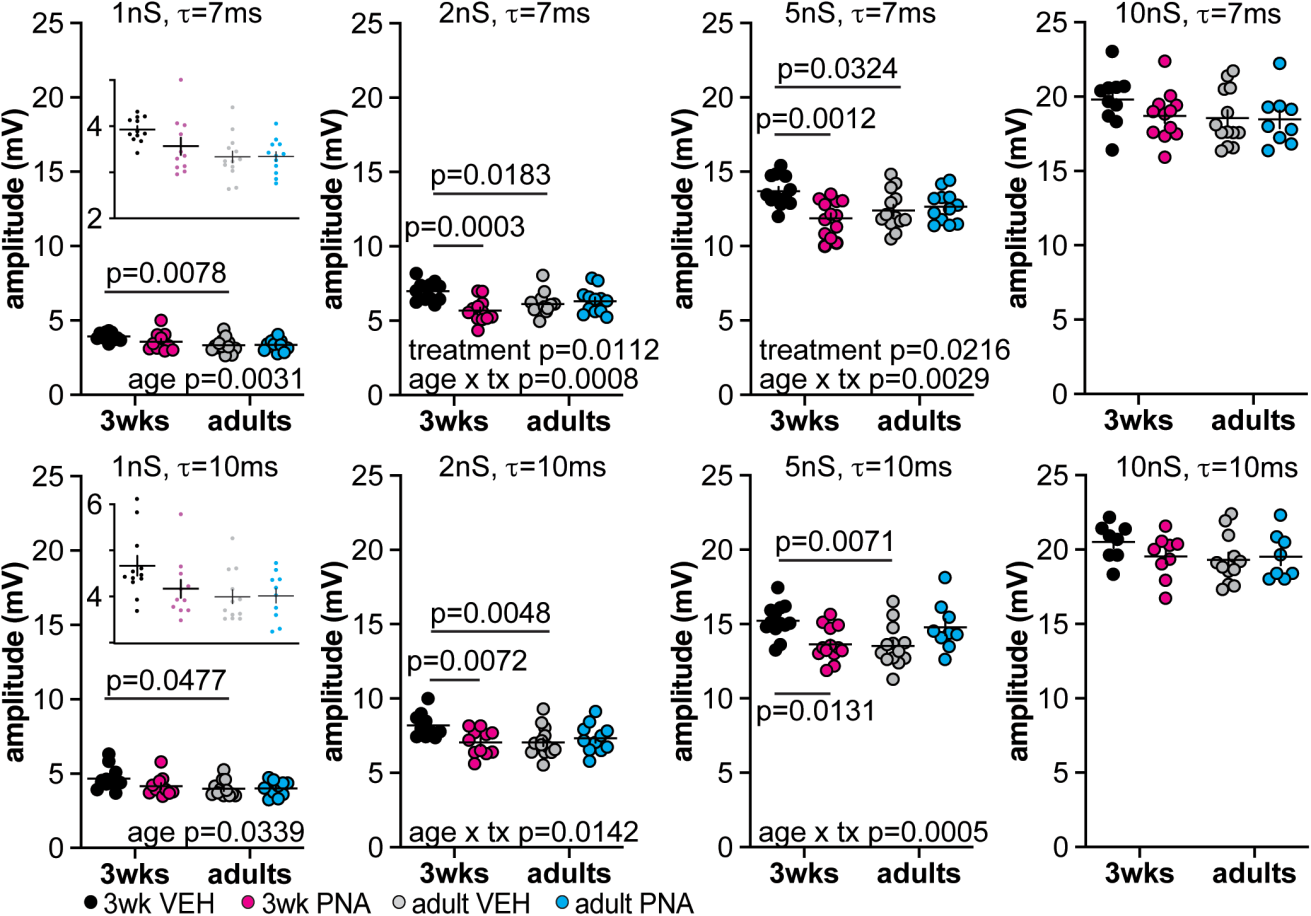
Amplitude of GnRH neuron dcPSPs. Individual values and mean ± SEM amplitude. Modeled conductances with a 7 ms decay time constant (*τ*) are in the top row and those with a 10 ms decay time constant are in the bottom row. Insets with expanded *y*‐axis in the left (1 nS) column are on the same scale in the upper and lower row but cover a different axis range. Two‐way ANOVA and *Šídák's* multiple comparison test parameters are in Table 9.

The amplitude of dcPSPs was mildly lower in adults at the smallest conductance tested with both decay‐time constants, again consistent with the developmental increase in capacitance (Figure 6, Table 8). The comparison of dcPSP amplitude in response to the middle conductances of 2 or 5 nS among groups is more nuanced. Specifically, whereas with the kinetic properties, most changes were primarily associated with development and consistent with capacitance changes, dcPSP amplitude was more affected by treatment and treatment by development interactions. Notably, cells from 3‐week‐old VEH mice had higher amplitude dcPSPs than either 3‐week‐old PNA mice or adult VEH mice. At the highest amplitude conductance, no differences in dcPSP amplitude were observed.

**TABLE 8:** Statistical parameters from two‐way ANOVA for dcPSP amplitudes (mV; Figure 6).

dcPSPs amplitude (mV; two‐way ANOVA)					
	Age	Treatment		Interaction	
dcGABA conductances (7 ms decay time constants)					
1 nS	Diff, 0.4063 [Cl, 0.1445, 0.6681] *F*(1,44) = 9.782; ***p* = 0.0031**	Diff, 0.1742 [Cl, −0.088, 0.4360] *F*(1,44) = 1.799; *p* = 0.1867		Diff, 0.3719 [Cl, −0.1517, 0.8955] *F*(1,44) = 2.049; *p* = 0.1594	
	*Šídák's* multiple comparisons	VEH 3 weeks versus VEH adults	PNA 3 weeks versus PNA adults	VEH 3 weeks versus PNA 3 weeks	VEH adults versus PNA adults
		***p* = 0.0078**	*p* = 0.6773	*p* = 0.2234	*p* > 0.9999
2 nS	Diff, 0.1131 [Cl, −0.3141, 0.5402] *F*(1,46) = 0.284; *p* = 0.5967	Diff, 0.5609 [Cl, 0.1338, 0.9881] *F*(1,46) = 6.987; ***p* = 0.0112**		Diff, 1.325 [Cl, 0.6742, 2.383] *F*(1,46) = 12.97; ***p* = 0.0008**	
	*Šídák's* multiple comparisons	VEH 3 weeks versus VEH adults	PNA 3 weeks versus PNA adults	VEH 3 weeks versus PNA 3 weeks	VEH adults versus PNA adults
		***p* = 0.0183**	*p* = 0.4406	***p* = 0.0003**	*p* = 0.9342
5 nS	Diff, 0.2508 [Cl, −0.4103, 0.9119] *F*(1,46) = 0.583; *p* = 0.4490	Diff, 0.7810 [Cl, 0.1199, 1.442] *F*(1,46) = 5.655; ***p* = 0.0216**		Diff, 2.065 [Cl, 0.7427, 3.387] *F*(1,46) = 9.882; ***p* = 0.0029**	
	*Šídák's* multiple comparisons	VEH 3 weeks versus VEH adults	PNA 3 weeks versus PNA adults	VEH 3 weeks versus PNA 3 weeks	VEH adults versus PNA adults
		***p* = 0.0324**	*p* = 0.3415	***p* = 0.0012**	*p* = 0.9720
10 nS	Diff, 0.7386 [Cl, −0.3528, 1.830] *F*(1,40) = 1.871; *p* = 0.1790	Diff, 0.5824 [Cl, −0.5090, 1.674] *F*(1,40) = 1.163; *p* = 0.2873		Diff, 1.001 [Cl, −1.182, 3.183] *F*(1,40) = 0.858; *p* = 0.3597	
dcGABA conductances (10 ms decay time constants)					
1 nS	Diff, 0.4116 [Cl, 0.033, 0.7903] *F*(1,41) = 4.816; ***p* = 0.0339**	Diff, 0.2407 [Cl, −0.1381, 0.6194] *F*(1,41) = 1.647; *p* = 0.2066		Diff, 0.5012 [Cl, −0.2365, 1.279] *F*(1,41) = 1.929; *p* = 0.1723	
	*Šídák's* multiple comparisons	VEH 3 weeks versus VEH adults	PNA 3 weeks versus PNA adults	VEH 3 weeks versus PNA 3 weeks	VEH adults versus PNA adults
		***p* = 0.0477**	*p* = 0.9702	*p* = 0.2278	*p* > 0.9999
2 nS	Diff, 0.4338 [Cl, −0.1148, 0.9823] *F*(1,42) = 2.546; *p* = 0.1180	Diff, 0.4338 [Cl, −0.1147, 0.9824] *F*(1,42) = 2.547; *p* = 0.1180		Diff, 1.391 [Cl, 0.2937, 2.488] *F*(1,42) = 6.545; ***p* = 0.0142**	
	*Šídák's* multiple comparisons	VEH 3 weeks versus VEH adults	PNA 3 weeks versus PNA adults	VEH 3 weeks versus PNA 3 weeks	VEH adults versus PNA adults
		***p* = 0.0048**	*p* = 0.5060	***p* = 0.0072**	*p* = 0.4817
5 nS	Diff, 0.2755 [Cl, −0.6019, 0.9313] *F*(1,44) = 0.5245; *p* = 0.4728	Diff, 0.1647 [Cl, −0.6019, 0.9313] *F*(1,44) = 0.1874; *p* = 0.6672		Diff, 2.844 [Cl, 1.311, 1.042] *F*(1,44) = 13.98; ***p* = 0.0005**	
	*Šídák's* multiple comparisons	VEH 3 weeks versus VEH adults	PNA 3 weeks versus PNA adults	VEH 3 weeks versus PNA 3 weeks	VEH adults versus PNA adults
		***p* = 0.0071**	*p* = 0.1792	***p* = 0.0131**	*p* = 0.1183
10 nS	Diff, 0.6176 [Cl, −0.4123, 1.647] *F*(1,33) = 1.489; *p* = 0.2311	Diff, 1.190 [Cl, −0.8692, 3.250] *F*(1,33) = 0.5982; *p* = 0.4448		Diff, 1.190 [Cl, −0.8692, 3.250] *F*(1,33) = 1.383; *p* = 0.2480	

*Note*: Bold indicates *p* < 0.05.

### 3.4 In silico analysis of the effects of capacitance

**FIGURE 7:**
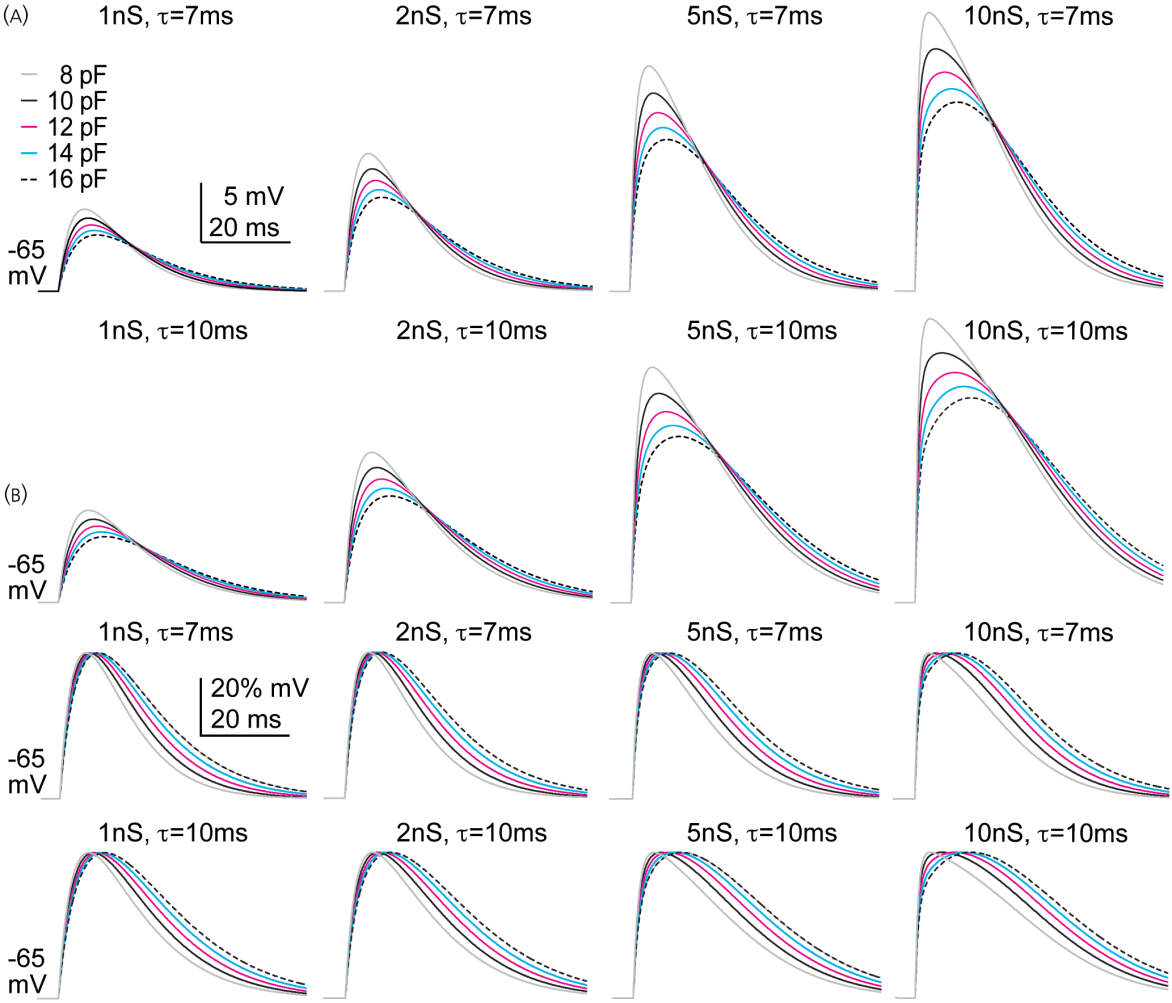
Effect of capacitance on shape of the potential generated with an RC circuit. Raw (A) and normalized (B) simulated postsynaptic potentials as a function of capacitance and synaptic conductance.

Several of the dcPSP properties quantified had apparent age‐dependent effects (3 week vs. adult). Because the capacitance of adult cells was higher than those recorded at 3 weeks, we did a simple resistor:capacitor model to see how varying capacitance affected the shape of dcPSPs. Input resistance was set to 737 MΩ, the average of all cells recorded, and capacitance was varied in 2 pF increments from 8 to 16 pF (Figure 7). This analysis suggests age effects on dcPSP parameters reported are likely attributable to the age‐related increase in capacitance.

### 3.5 Action potential GnRH neuron responses to 5 nS simulated GABA conductance

No cells in any group fired in response to 1 or 2 nS simulated conductances. Five of 15 neurons from 3‐week‐old VEH females fired in response to the 5 nS, 7 ms decay time constant conductance; this response was observed in 2–10 of the 10 traces tested and there was no effect of trace order. No cells from the other experimental groups generated action potentials in response to this conductance. When the decay time constant of the 5 nS conductance was increased to 10 ms, six out of 15 cells from 3‐week‐old VEH females generated action potentials. In contrast, only one of 13 cells from VEH adults and 2 of 10 cells from PNA adult females generated action potentials. None of the 13 cells from 3‐week‐old PNA females generated action potentials in response to the 5 nS conductance. Because only cells from 3‐week‐old VEH females fired sufficiently for even rudimentary statistical analyses, we compared properties of action potentials generated with the two different decay time constants. No differences were found in any property examined (all *p* < 0.13 unpaired *t*‐test with Welch's correction; Table 9).

**TABLE 9:** Descriptive statistics of the action potential properties measured in response to the 5 nS in 3‐week VEH mice, comparing 7 ms and 10 ms decay time constant.

Property	7 ms decay time constant (*n* = 5)	10 ms decay time constant (*n* = 6)	Welch's *t*‐test parameters
Latency (ms)	8.61 ± 1.02	9.05 ± 1.14	*p* = 0.7841, *t* = 0.2823, df = 9.000
AP threshold (mV)	−46.54 ± 1.31	−44.65 ± 1.26	*p* = 0.3433, *t* = 1.003, df = 8.375
Rate of rise (mV/ms)	0.43 ± 0.02	0.42 ± 0.02	*p* = 0.7550, *t* = 0.2953, df = 8.375
AP amplitude (mV)	80.76 ± 1.61	79.97 ± 1.17	*p* = 0.7030, *t* = 0.3959, df = 7.647
FWHM (ms)	0.71 ± 0.02	0.69 ± 0.02	*p* = 0.4717, *t* = 0.7518, df = 8.820
AHP amplitude (mV)	−24.90 ± 0.65	−25.60 ± 1.22	*p* = 0.6263, *t* = 0.5077, df = 7.486
AHP time (ms)	2.85 ± 0.11	2.60 ± 0.10	*p* = 0.1390, *t* = 1.632, df = 8.548

### 3.6 Action potential GnRH neuron responses to 10 nS simulated GABA conductance

**FIGURE 8:**
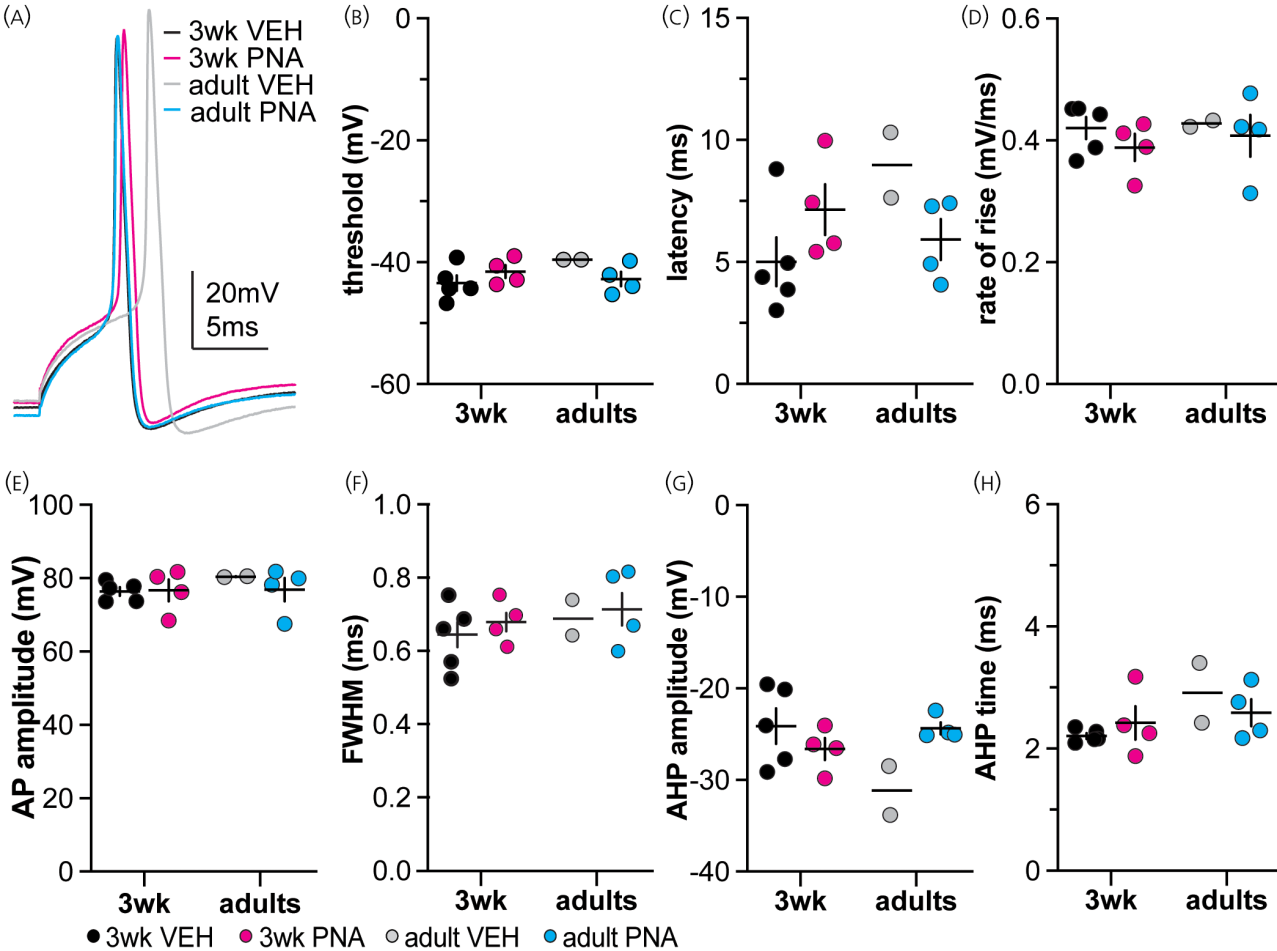
Averaged action potentials induced by the 10 nS, 7 ms decay time constant conductance. (A) Representative traces from each group. (B–H) Cell‐level mean ± SEM for threshold (B), latency (C), rate of rise (D), amplitude (E), full width at half maximum (FWHM, F), amplitude of the afterhyperpolarization potential (AHP, G) and time of the AHP peak (H).

**FIGURE 9:**
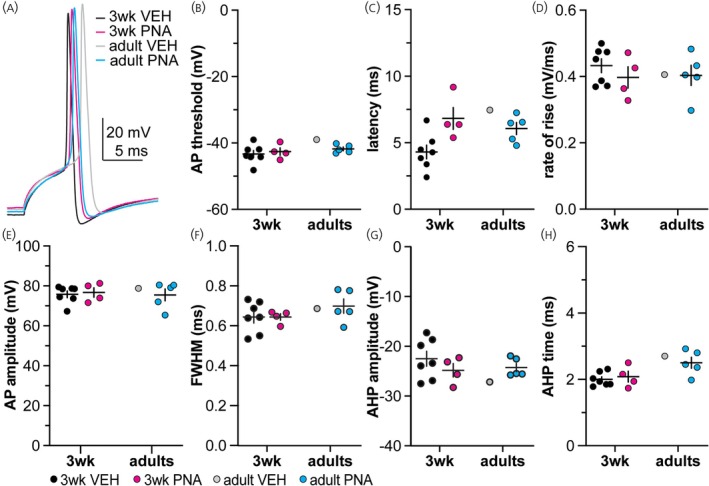
Averaged action potentials induced by the 10 nS, 10 ms decay time constant conductance. (A) Representative traces from each group. (B–H) Cell‐level mean ± SEM for threshold (B), latency (C), rate of rise (D), amplitude (E), full width at half maximum (FWHM, F), amplitude of the afterhyperpolarization potential (AHP, G) and time of the AHP peak (H).

Figures 8A and 9A show representative traces of action potentials for the 7 ms and 10 ms decay time constant, respectively. Because only one or two cells from adult VEH mice fired, statistical comparisons were not done but data are shown in Figures 8B–H and 9B–H and Tables 5, 10 and 11. Qualitative observations include more cells fired action potentials in response to the 10 nS simulated GABA conductance tested (29.9%; 32 of 107 cells) than the 5 nS conductance (13.6%, 14 of 103 cells) regardless of decay time constant. Cells from adult VEH control mice were the least likely to fire (11.5%) in contrast to cells from 3‐week‐old VEH mice, which were the most likely to fire (41.4%).

**TABLE 10:** Descriptive statistics of the action potentials properties measured in response to the 10 nS, 7 ms decay time constant, conductance and statistical comparisons of the action potentials between 3‐week‐old VEH and PNA females (Figure 8).

Descriptive statistics (mean ± SEM when possible)					
Property	VEH 3 weeks	PNA 3 weeks	VEH adults	PNA adults	PNA 3 weeks (delayed spike)
Latency (ms)	4.530 ± 0.320	6.632 ± 0.395	8.517 ± 0.911	5.679 ± 0.286	11.78 ± 0.4250
AP threshold (mV)	−44.032 ± 0.404	−41.539 ± 0.379	−39.575 ± 0.197	−43.361 ± 0.321	−51.37 ± 0.2009
Rate of rise (mV/ms)	0.419 ± 0.007	0.376 ± 0.009	0.429 ± 0.008	0.433 ± 0.009	0.4020 ± 0.0038
AP amplitude (mV)	79.067 ± 0.394	74.922 ± 1.136	80.428 ± 0.211	79.170 ± 0.692	89.44 ± 0.3447
FWHM (ms)	0.655 ± 0.011	0.669 ± 0.002	0.674 ± 0.027	0.694 ± 0.015	0.7130 ± 0.0183
AHP amplitude (mV)	−23.513 ± 0.679	−26.874 ± 0.495	−32.041 ± 1.798	−24.329 ± 0.262	−21.43 ± 0.1888
AHP time (ms)	2.200 ± 0.026	2.320 ± 0.099	2.750 ± 0.035	2.461 ± 0.064	2.425 ± 0.2750

Statistical parameters comparing the action potential properties between 3‐week‐old VEH and PNA females				
Property	VEH 3 weeks (median ± IQR)	PNA 3 weeks (median ± IQR)	Mann–Whitney *U* test	Two‐tailed *p*‐value
Latency (ms)	4.386	6.602	*U* = 3	0.1111
	(3.442 to 6.875)	(5.507 to 9.330)		
AP threshold (mV)	−44.28	−41.72	*U* = 5	0.2857
	(−45.51 to −39.38)	(−43.46 to −39.38)		
Rate of rise (mV/ms)	0.4426	0.4006	*U* = 6	0.4127
	(0.3771 to 0.4520)	(0.3415 to 0.4231)		
AP amplitude (mV)	77.29	78.26	*U* = 8	0.7302
	(73.68 to 78.66)	(70.40 to 81.35)		
FWHM (ms)	0.6622	0.6769	*U* = 7	0.5556
	(0.5680 to 0.7117)	(0.6314 to 0.7276)		
AHP amplitude (mV)	−24.05	−26.30	*U* = 6	0.4127
	(−28.42 to −19.83)	(−29.00 to −24.57)		
AHP time (ms)	2.163	2.317	*U* = 7	0.5556
	(2.121 to 2.313)	(1.971 to 2.977)		

*Note*: Bold indicates *p* < 0.05.

**TABLE 11:** Descriptive statistics of action potential properties and statistical parameters comparing the action potentials of GnRH neurons in response to the 10 nS, 10 ms decay time constant, conductance between 3‐week‐old VEH and PNA females (Figures 9 and 10).

Descriptive statistics (mean ± SEM when possible)					
Property	VEH 3 weeks	PNA 3 weeks	VEH adults	PNA adults	PNA 3 weeks (delayed spike)
Latency (ms)	4.25 ± 0.19	6.64 ± 0.28	7.45 ± 0.00	5.79 ± 0.17	12.86 ± 0.87
AP threshold (mV)	−43.49 ± 0.41	−42.54 ± 0.43	−38.96 ± 0.00	−42.08 ± 0.20	−45.77 ± 0.47
Rate of rise (mV/ms)	0.43 ± 0.01	0.40 ± 0.01	0.41 ± 0.00	0.42 ± 0.01	0.22 ± 0.05
AP amplitude (mV)	75.73 ± 0.63	76.80 ± 0.80	78.77 ± 0.00	77.30 ± 0.99	60.18 ± 4.43
FWHM (ms)	0.65 ± 0.01	0.65 ± 0.004	0.69 ± 0.00	0.68 ± 0.01	1.08 ± 0.08
AHP amplitude (mV)	−22.13 ± 0.56	−25.12 ± 0.48	−27.14 ± 0.00	−24.38 ± 0.32	−16.57 ± 1.19
AHP time (ms)	2.01 ± 0.03	2.06 ± 0.06	2.70 ± 0.00	2.37 ± 0.06	4.52 ± 0.45

Statistical parameters comparing the action potential properties between 3‐week‐old VEH and PNA females				
Property	VEH 3 weeks (median ± IQR)	PNA 3 weeks (median ± IQR)	Mann–Whitney *U* test	Two‐tailed *p*‐value
Latency (ms)	4.275	6.374	*U* = 3	**0.0424**
	(3.379 ± 5.083)	(5.632 ± 8.479)		
AP threshold (mV)	−43.83	−42.83	*U* = 13	0.9273
	(−44.21 to −401.91)	(−44.55 to −40.42)		
Rate of rise (mV/ms)	0.45	0.39	*U* = 7	0.2303
	(0.37 ± 0.48)	(0.34 ± 0.46)		
AP amplitude (mV)	78.47	76.97	*U* = 11	0.6485
	(73.71 ± 78.74)	(72.19 ± 80.94)		
FWHM (ms)	0.643	0.656	*U* = 14	>0.9999
	(0.55 ± 0.719)	(0.61 ± 0.67)		
AHP amplitude (mV)	−23.36	−24.36	*U* = 10	0.5273
	(−26.88 to −18.66)	(−27.58 to −22.49)		
AHP time (ms)	1.938	2.05	*U* = 12	0.7879
	(1.85 ± 2.24)	(1.79 ± 2.41)		

Statistical parameters comparing the AP properties of normal spikes vs delayed spikes (PNA 3 weeks)				
Property	PNA 3 weeks NORMAL spike (median ± IQR)	PNA 3 weeks DELAYED spike (median ± IQR)	Mann–Whitney *U* test	Two‐tailed *p*‐value
Latency (ms)	6.10	12.10	*U* = 3	**<0.0001**
	(5.50 to 7.65)	(11.58 to 14.83)		
AP threshold (mV)	−43.14	−45.70	*U* = 28	**0.0003**
	(−44.78 to −40.09)	(−46.87 to −44.52)		
Rate of rise (mV/ms)	0.41	0.15	*U* = 45	**0.0040**
	(0.35 to 0.44)	(1.14 to 0.30)		
AP amplitude (mV)	77.94	54.09	*U* = 52	**0.0098**
	(72.94 to 80.49)	(52.20 to 69.61)		
FWHM (ms)	0.66	1.171	*U* = 10	**<0.0001**
	(0.62 to 0.66)	(0.91 to 1.21)		
AHP amplitude (mV)	−25.40	−15.12	*U* = 12	**<0.0001**
	(−26.23 to −23.16)	(−18.45 to −14.48)		
AHP time (ms)	2.05	5.00	*U* = 13	**<0.0001**
	(1.85 to 2.20)	(3.43 to 5.70)		

*Note*: Bold indicates *p* < 0.05.

**FIGURE 10:**
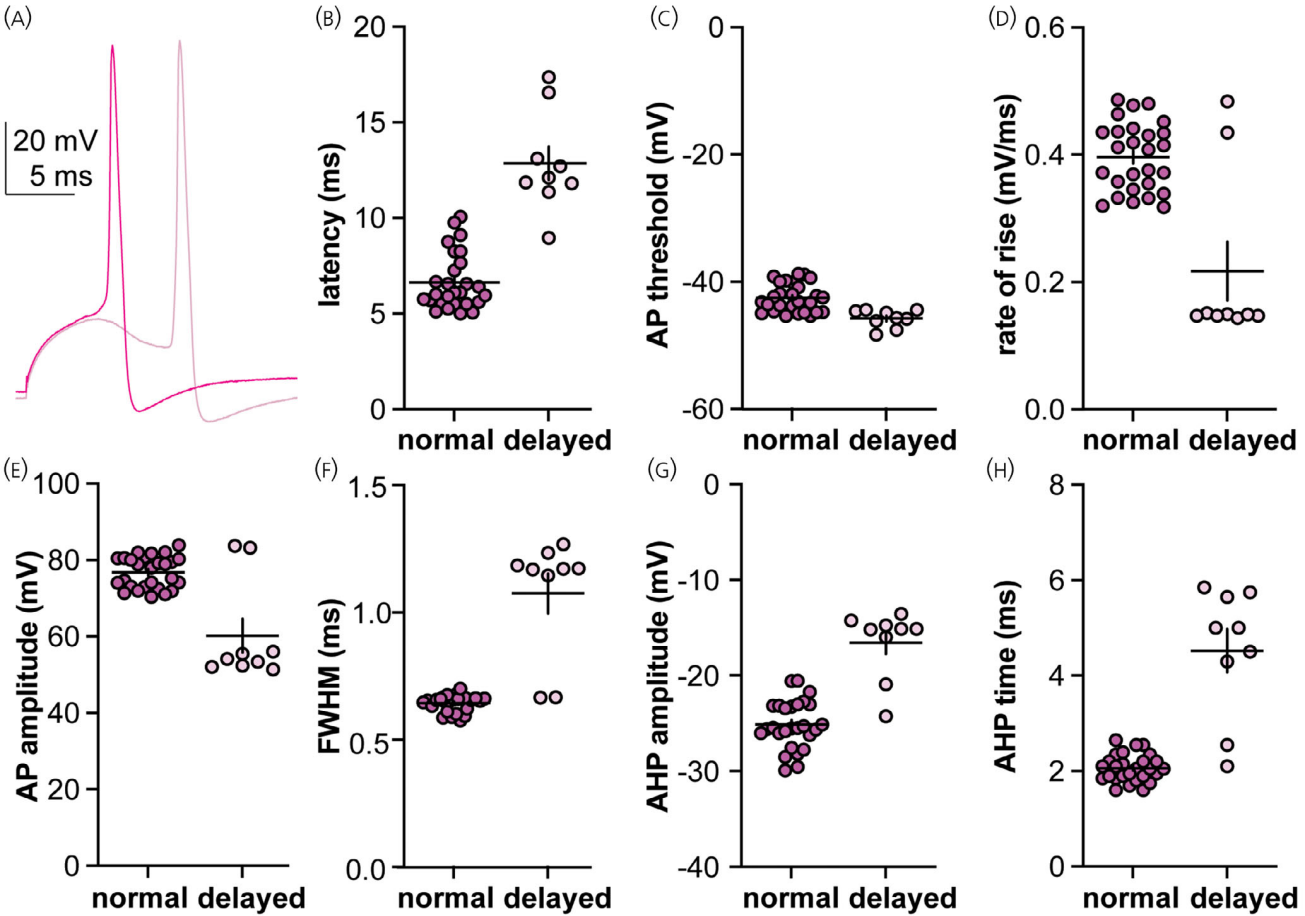
Delayed action potential generation observed only in GnRH neurons from 3‐week‐old PNA mice. (A) Representative traces of a normal and delayed action potential from the same cell in response to the 10 nS, 10 ms decay time conductance. (B–H) Spike‐level individual values and mean ± SEM latency (B), threshold (C), rate of rise (D), amplitude (E), full width at half maximum (FWHM, F), amplitude of the afterhyperpolarization potential (AHP, G) and time of the AHP peak (H) in response to the 10 nS, 10 ms decay time constant conductance.

Delayed action potentials (“delayed spikes”) were detected in a subset of recordings from 3‐week‐old PNA females. Data from the 10 ms decay time constant conductances are shown in Figure 10. Only one cell exhibited a delayed spike using the 7 ms decay time constant, whereas two cells responded with delayed spikes using the 10 ms decay time constant (of these, one also fired typical action potentials). Delayed spikes occurred after the peak of the dcPSP. This was an observation that was exclusive to the 3‐week‐old PNA group, making it interesting, but given no formal design statistical analysis is likely inappropriate and the data are presented for information only. These spikes had an apparently greater latency, hyperpolarized threshold, lower rate of rise, lower amplitude, larger FWHM, smaller AHP and longer time to the peak of the AHP than non‐delayed spikes (Figure 10B–H, Table 11). The threshold potential (defined as second derivative >10,000 V/s/s) appeared hyperpolarized relative to non‐delayed spikes and was hyperpolarized relative to the peak of the dcPSP in each case.

## 4 DISCUSSION

The dynamic secretion patterns of GnRH/LH throughout the female reproductive cycle play a critical role in maintaining reproduction. Disruptions in these patterns of secretion, such as observed in hyperandrogenemic PCOS patients, contribute to impaired fertility. Here, we used dynamic clamp to simulate the same physiologic GABAergic conductances to GnRH neurons from 3‐week‐old and adult VEH and PNA females to measure how differences in intrinsic properties of GnRH neurons shape their response to the synaptic input.

Of the dcPSP properties analyzed, amplitude exhibited the most notable difference with PNA treatment. This was observed at the intermediate conductances tested for both decay time constants. The two decay time constants tested reflected developmental differences in this parameter but the pattern of amplitude responses was the same. Specifically, at 2 and 5 nS conductances, cells from 3‐week‐old VEH mice had larger dcPSPs than cells from 3‐week‐old PNA and adult VEH mice. The reduced dcPSP amplitude in 3‐week‐old PNA versus VEH mice may be attributed to greater activation of subthreshold voltage‐gated potassium channels countering further membrane depolarization^36^ in PNA mice. Density of the transient potassium current is greater in 3‐week‐old PNA than 3‐week‐old VEH mice.^25^ Consistent with this, 3‐week‐old PNA females have a blunted action potential response to local GABA application than 3‐week‐old VEH females.^21^ There was a developmental decrease in dcPSP amplitude in VEH mice. dcPSPs with action potentials are not included due to complications in detecting the peak of the dcPSP in the presence of an action potential, potentially excluding larger amplitude dcPSPs from this comparison. The largest dcPSP amplitude was observed in the group with the most frequent initiation of action potentials (3‐week‐old VEH); however, suggesting the developmental decrease in dcPSP amplitude is driven by something else. One possibility is the hyperpolarized voltage‐dependence of activation of the transient‐subthreshold potassium current in VEH adults, which may counter membrane depolarization.^25^

Interestingly, there was no difference in amplitude among groups at the largest conductance tested. While this might be in part attributable to the increased number of dcPSPs initiating action potentials, being excluded and thus reducing power as mentioned above, the larger depolarization generated by this conductance may overcome any differential activation of currents among groups observed with more subtle depolarizations. How our test conductances compare to the properties of GABAergic inputs to GnRH neurons in vivo remain unknown despite modeling synaptic conductances from recordings using physiologic chloride levels. The observed shifts in dcPSP amplitude nonetheless suggest GnRH neurons from VEH mice are poised to be more responsive to synaptic input before puberty. Indeed, the highest conductances tested generated more action potentials in prepubertal controls. It is important to point out that using dynamic clamp to simulate conductances precludes changes in transmitter release or postsynaptic receptor composition as contributors to any changes in dcPSP properties. Together, these observations suggest both age and PNA treatment can alter GnRH neuron output as defined by action potential firing. These observations are consistent with previous data in which GnRH neurons from 3‐week‐old VEH females have the highest spontaneous firing activity of these groups.^20^

The reduced action potential response of adult GnRH neurons in the present study contrasts with a previous study using the same animal models but a different electrophysiologic approach.^24^ Using traditional depolarizing current‐clamp steps (0.5 s duration) to examine excitability, GnRH neurons from adult females were more excitable, defined by the number of action potentials at a given current injection step, than neurons from 3‐week‐old females, regardless of PNA treatment.^24^ There are at least three possible explanations for this. First, dynamic‐clamp, which simulates a single synaptic conductance, provides a more physiologic stimulus; that is, the current injected with dynamic clamp is modified as the targeted cell depolarizes, whereas the current is constant during the stepwise stimulus. Interestingly, the properties of the dynamic‐clamp‐induced action potentials of the present study are similar to the first action potential at rheobase in previous work,^24^ despite a refinement in the method for calculating threshold. Second, the initial membrane potential was more depolarized in the present study. While groups being compared did not differ, the more depolarized condition is expected to increase the inactivation of voltage‐dependent channels. Third, during the prolonged current steps of the prior study, GnRH neurons generated multiple action potentials and there was only a 1 s interval between the subsequent current injections.^24^ In the present study, there was at minimum a 2.5 s interval between test conductances. Differences in the timing between stimuli across the two experimental approaches could affect the response of the GnRH neurons from one stimulus to the next if the underlying ionic conductances have insufficient time to recover. Finally, the age‐related increase in capacitance observed in the present study was a trend in the prior work that did not reach the level accepted for significance. *In silico* analysis demonstrated that the effects of age on dcPSP parameters detected can be mimicked solely by an increase in capacitance. PNA treatment had no effect on capacitance, suggesting its effects are independent.

An intriguing finding was the delayed initiation of action potentials that occurred in a subset of GnRH neurons from 3‐week‐old PNA mice. Relating this directly to GnRH/LH release dynamics is precluded by the low blood volume of 3‐week‐old mice. GnRH neurons in the 3‐week‐old PNA group do, however, have a lower spontaneous firing rate^20^ despite increased GABAergic input compared to cells from VEH mice at this age, and a reduced response to locally applied GABA.^21^ This suggests the intrinsic properties of GnRH neurons in the PNA group have responded with changes that maintain lower firing rate^20^ despite the PNA‐induced increase in GABAergic input at this age. This includes the above‐mentioned increased transient potassium current density in cells from 3‐week‐old PNA versus VEH mice.^25^ This current might also contribute to the delayed initiation of action potentials in some cells from this group in the present study. It is important to note that all recordings and the application of the dynamic‐clamp stimulus were done at the GnRH neuron soma.

Differences in the action potential waveforms described above could also be attributed to differences in the location of action potential initiation or changes in axial resistance occurring along the GnRH neuron processes. The length of the axon initial segment is dynamic during development, tending to be longer in embryonic and preweaning mice,^37^, ^38^ and can be modified by drug exposure and/or changes in the environment.^39^ Attempts to identify an axon initial segment (AIS) in GnRH neurons using staining for ankyrin G were not conclusive.^40^ Immunohistochemical staining for voltage‐gated sodium channels shows their expression along the length of GnRH neuron processes, but detected no concentration in any subcellular domain; this was done without staining for Ankyrin G or other AIS markers, thus it remains unclear whether there is co‐expression of sodium channels or a specialized AIS compartment in these neurons.^41^ Of interest in this regard, one paper suggests GnRH neurons can initiate action potentials in either the somatic or dendritic region.^41^ In other neuron types, such as in dissociated embryonic hippocampal neurons (12–14 days in vitro), changes in the AIS location as a result of changes in action potential activity have been correlated with changes in excitability in response to controlled current injections, suggesting that the AIS is susceptible to activity‐dependent plasticity.^42^ It thus seems possible that the location(s) of action potential initiation in GnRH neurons from 3‐week‐old PNA females might undergo changes as a result of the altered spontaneous firing activity at this developmental period.^20^ Another possible explanation is that the expression and/or function of the voltage‐gated ion channels in different GnRH neuron subcompartments are altered with PNA treatment at 3 weeks of age and that these changes are what give rise to the delayed action potential recorded at the soma. The delayed spike was observed in only a subset of cells in this group and, at present, these studies are underpowered. Increasing the number of GnRH neuron recordings will clarify if this form of action potential is unique to prepubertal PNA mice.

A growing body of work indicates PNA treatment alters synaptic and intrinsic properties of GnRH neurons. The synaptic changes appear to be maintained from a prepubertal to adult state in cells from PNA mice, whereas some intrinsic changes show development‐dependent modification. This includes the above‐mentioned changes in properties of two types of voltage‐dependent potassium currents in GnRH neurons.^25^ Further, unpublished data from our lab indicate the properties of voltage‐gated calcium currents are altered with both age and PNA treatment and these currents could contribute to the differences we have observed. Age as well as PNA‐related changes in sodium channels or organization of an AIS could also be occurring. At this point, however, little is known about the functional role these currents play in shaping GnRH neuron activity, but future work can utilize dynamic clamp to address how specific intrinsic properties shape GnRH neuron activity. Regardless, the preliminary findings in this study suggest that the response of GnRH neurons to the *same* physiological simulated GABA conductances are altered with age and PNA. Thus, it seems likely that the changes GnRH neurons are undergoing in this model contribute to previous observations, including the development of a PCOS‐like phenotype.

## AUTHOR CONTRIBUTIONS

Jennifer Jaime and Suzanne M. Moenter designed the research. Jennifer Jaime performed the research and analyzed the data. Jennifer Jaime and Suzanne M. Moenter wrote the paper. R. Anthony DeFazio helped with setup of the dynamic clamp system, modeling of the GABAergic conductance templates, editing of this manuscript, and performed the in silico analysis.

## FUNDING INFORMATION

This study was supported by National Institute of Health (NIH)/Eunice Kennedy Shriver National Institute of Child Health and Human Development Grant R01HD104345 (to Suzanne M. Moenter). Support for Jennifer Jaime was provided by NIH T32HD079342 and F31HD110279.

## CONFLICT OF INTEREST STATEMENT

The authors declare no conflicts of interest.

## Data Availability

The data that support the findings of this study are available from the corresponding author upon reasonable request.
